# Supplementary material for: McIdas localizes to centrioles and controls centriole numbers through PLK4-dependent phosphorylation
Source: EMBO Rep. 2026 Feb 5;27(6):1478–509. doi: 10.1038/s44319-026-00697-5 (PMC13022133; doi:10.1038/s44319-026-00697-5)
Supplement: Supplementary file 1 — Table EV1 [file 44319_2026_697_MOESM1_ESM.docx]

**Table EV1**

List of siRNA oligos

| **Target** | **siRNA oligos sequence** |
| --- | --- |
| McIdas siRNA_1 | 5’-CCACCAAACGGAAGCAGACTTCAAT-3’  5’-CCAAACAGGAACGGACATTCCCAAT-3’ |
| Luciferase siRNA | 5’-CGTACGCGGAATACTTCGA-3’ |
