# Supplementary material for: McIdas localizes to centrioles and controls centriole numbers through PLK4-dependent phosphorylation
Source: EMBO Rep. 2026 Feb 5;27(6):1478–509. doi: 10.1038/s44319-026-00697-5 (PMC13022133; doi:10.1038/s44319-026-00697-5)
Supplement: Supplementary file 2 — Table EV2 [file 44319_2026_697_MOESM2_ESM.docx]

**Table EV2**

List of primers

| **Set of primers** | **Primer sequence** |
| --- | --- |
| **For quantitative RT-PCR** | |
| hMcIdas_Fw | 5’-GACGCGCTTGTTGAGAATAA-3’ |
| hMcIdas_Rv | 5’-CACGTTCCGCTCCTTGAG-3’ |
| hFoxj1_Fw | 5’-ACGGACAACTTCTGCTACTTC-3’ |
| hFoxj1_Rv | 5’-TTGTTCAGAGACAGGTTGTGG-3’ |
| hcMyb_Fw | 5’-GAAGGTCGAACAGGAAGGTTATCT-3’ |
| hcMyb_Rv | 5’-GTAACGCTACAGGGTATGGAACA-3’ |
| hPLK4_Fw | 5’-ACCTGCATCGGGGAGAAGAT-3’ |
| hPLK4_Rv | 5’-TTCCTGCTTTGTACATGGCTTTC-3’ |
| hSAS6_Fw | 5’-CCAGCAGCAACACAATCAGAA-3’ |
| hSAS6_Rv | 5’-CCACAGGTTGGGTAAGTCAGTCT-3’ |
| hYWHAZ_Fw | 5’-GATCCCCAATGCTTCACAAG-3’ |
| hYWHAZ_Rv | 5’-TGCTTGTTGTGACTGATCGAC-3’ |
| **For the amplification of McIdas C-terminal region** | |
| Fw | 5’-GGAATTCCATATGACCCGTCCGGGTAACC-3’ |
| Rv | 5’-CCGGCTCGAGGCTCGGCACCCAACGGAACTT-3’ |
