# Supplementary figures and images for: McIdas localizes to centrioles and controls centriole numbers through PLK4-dependent phosphorylation
Source: EMBO Rep. 2026 Feb 5;27(6):1478–509. doi: 10.1038/s44319-026-00697-5 (PMC13022133; doi:10.1038/s44319-026-00697-5)

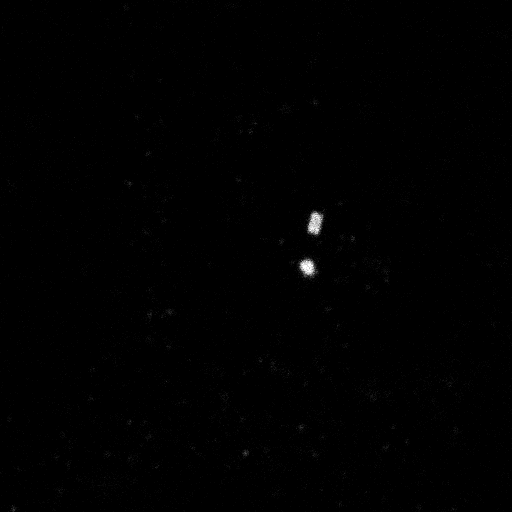

Supplement: Supplementary file 4 — Source data Fig. 1 [file 44319_2026_697_MOESM4_ESM.zip › Figure 1/1H/ACT_McIdas_G1_2.tif]

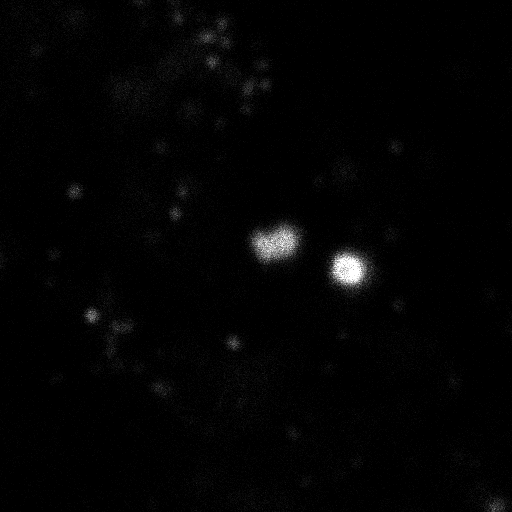

Supplement: Supplementary file 4 — Source data Fig. 1 [file 44319_2026_697_MOESM4_ESM.zip › Figure 1/1H/ACT_McIdas_G1_1.tif]

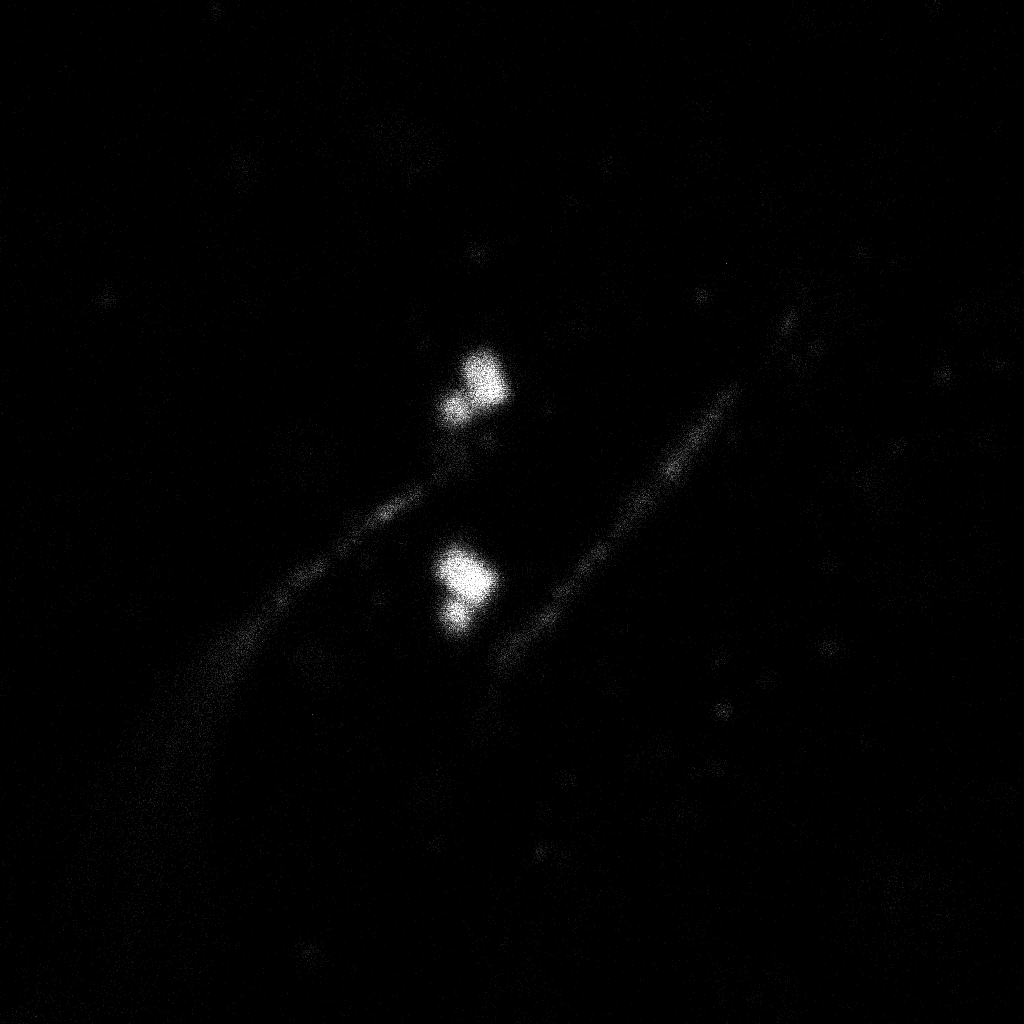

Supplement: Supplementary file 4 — Source data Fig. 1 [file 44319_2026_697_MOESM4_ESM.zip › Figure 1/1H/ACT_McIdas_S:G2.tif]

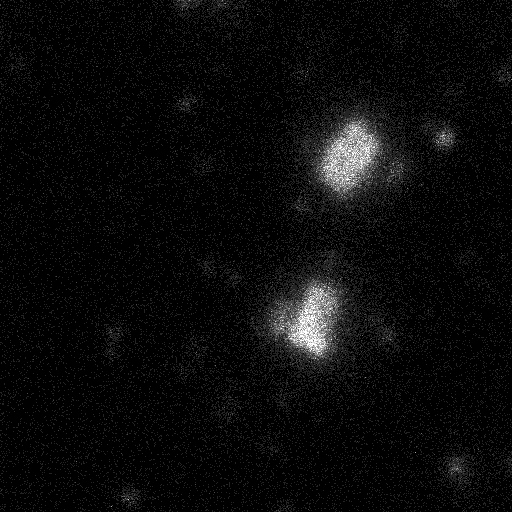

Supplement: Supplementary file 4 — Source data Fig. 1 [file 44319_2026_697_MOESM4_ESM.zip › Figure 1/1H/ACT_McIdas_S.tif]

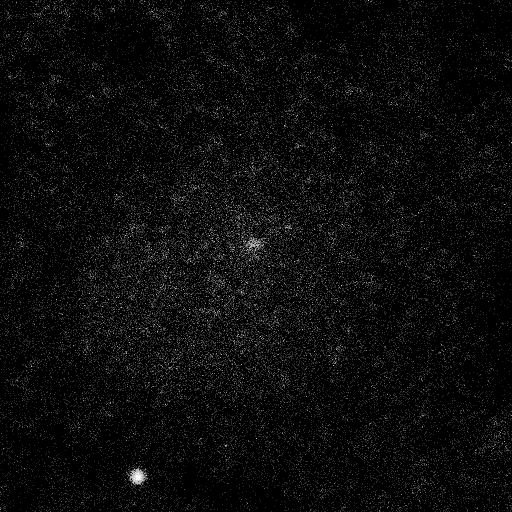

Supplement: Supplementary file 4 — Source data Fig. 1 [file 44319_2026_697_MOESM4_ESM.zip › Figure 1/1A/Centrin_McIdas_Dapi_EdU_Early S.tif]

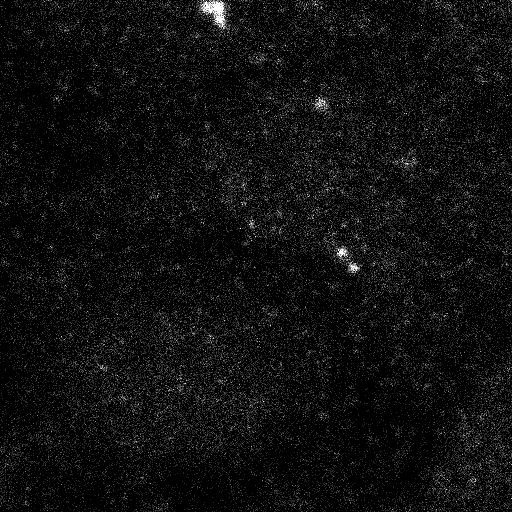

Supplement: Supplementary file 4 — Source data Fig. 1 [file 44319_2026_697_MOESM4_ESM.zip › Figure 1/1A/Centrin_McIdas_Dapi_EdU_Middle:late S.tif]

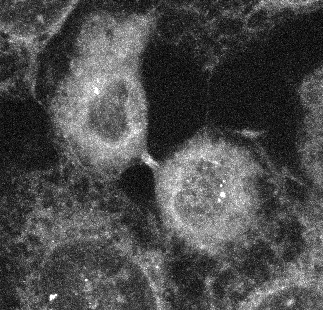

Supplement: Supplementary file 4 — Source data Fig. 1 [file 44319_2026_697_MOESM4_ESM.zip › Figure 1/1A/Centrin_McIdas_Dapi_EdU_Telophase.tif]

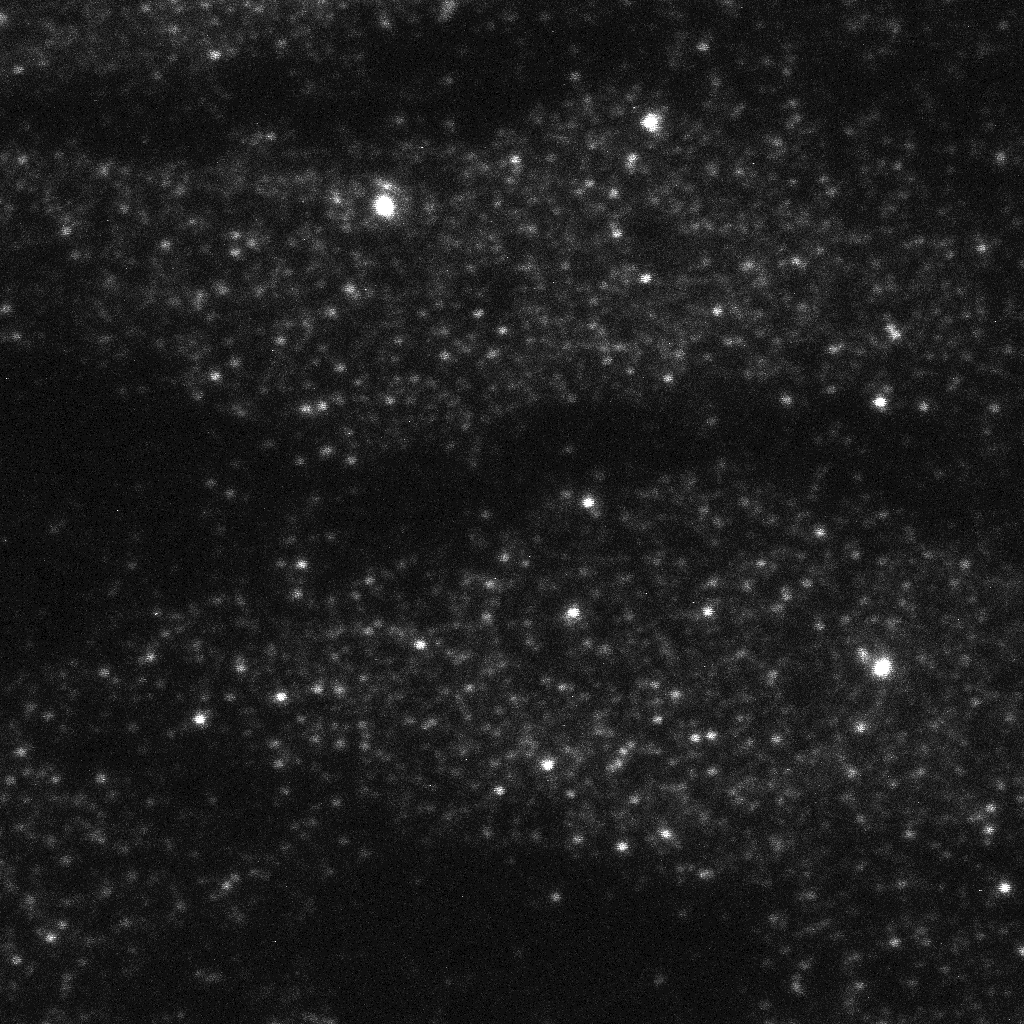

Supplement: Supplementary file 4 — Source data Fig. 1 [file 44319_2026_697_MOESM4_ESM.zip › Figure 1/1E/Cep164_Cep135_Dapi_McIdas.tif]

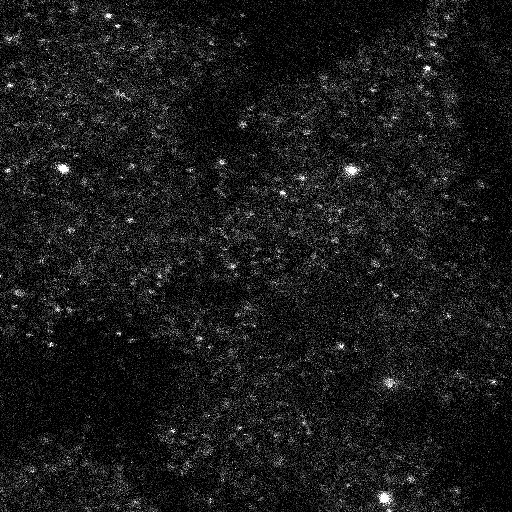

Supplement: Supplementary file 4 — Source data Fig. 1 [file 44319_2026_697_MOESM4_ESM.zip › Figure 1/1D/Cep164_McIdas_Dapi_Centrin.tif]

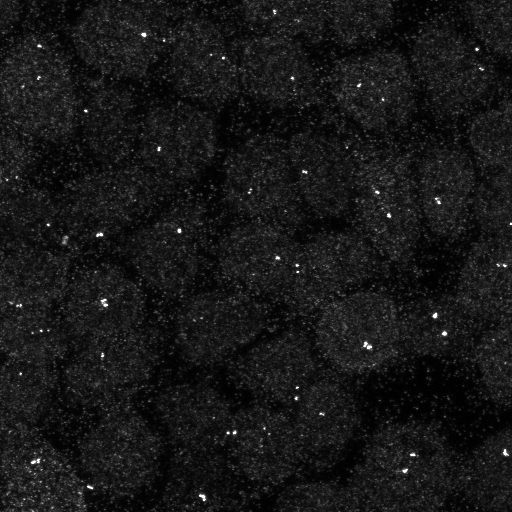

Supplement: Supplementary file 4 — Source data Fig. 1 [file 44319_2026_697_MOESM4_ESM.zip › Figure 1/1C/Centrin_GFP_Dapi_McIdas_HaLa GFP-McIdas.tif]

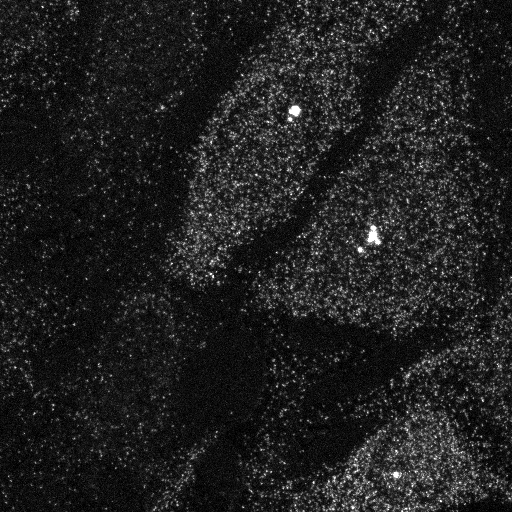

Supplement: Supplementary file 5 — Source data Fig. 2 [file 44319_2026_697_MOESM5_ESM.zip › Figure 2/2G/Dapi_SAS6_Luciferase siRNA.tif]

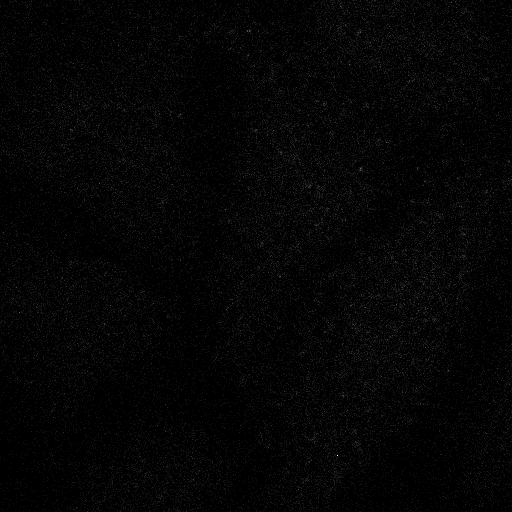

Supplement: Supplementary file 5 — Source data Fig. 2 [file 44319_2026_697_MOESM5_ESM.zip › Figure 2/2G/Dapi_SAS6_McIdas siRNA.tif]

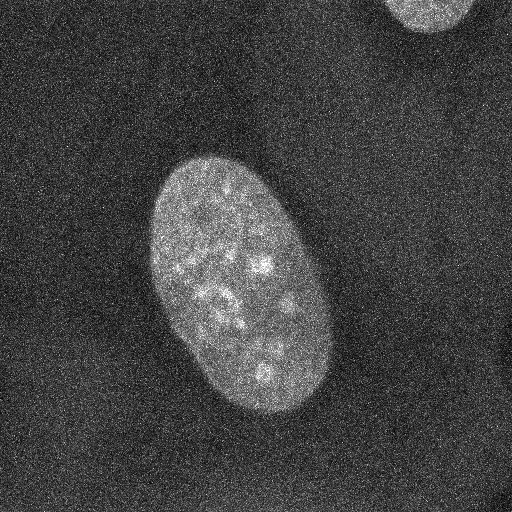

Supplement: Supplementary file 5 — Source data Fig. 2 [file 44319_2026_697_MOESM5_ESM.zip › Figure 2/2A/Cep135_GFP_Dapi_CP110_GFP.tif]

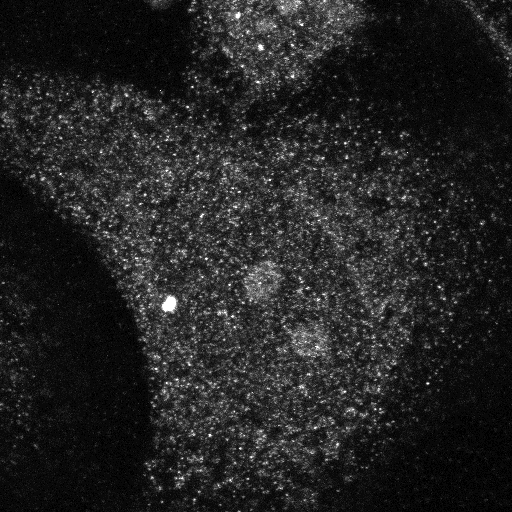

Supplement: Supplementary file 5 — Source data Fig. 2 [file 44319_2026_697_MOESM5_ESM.zip › Figure 2/2A/Cep135_GFP_Dapi_CP110_GFP-McIdas WT.tif]

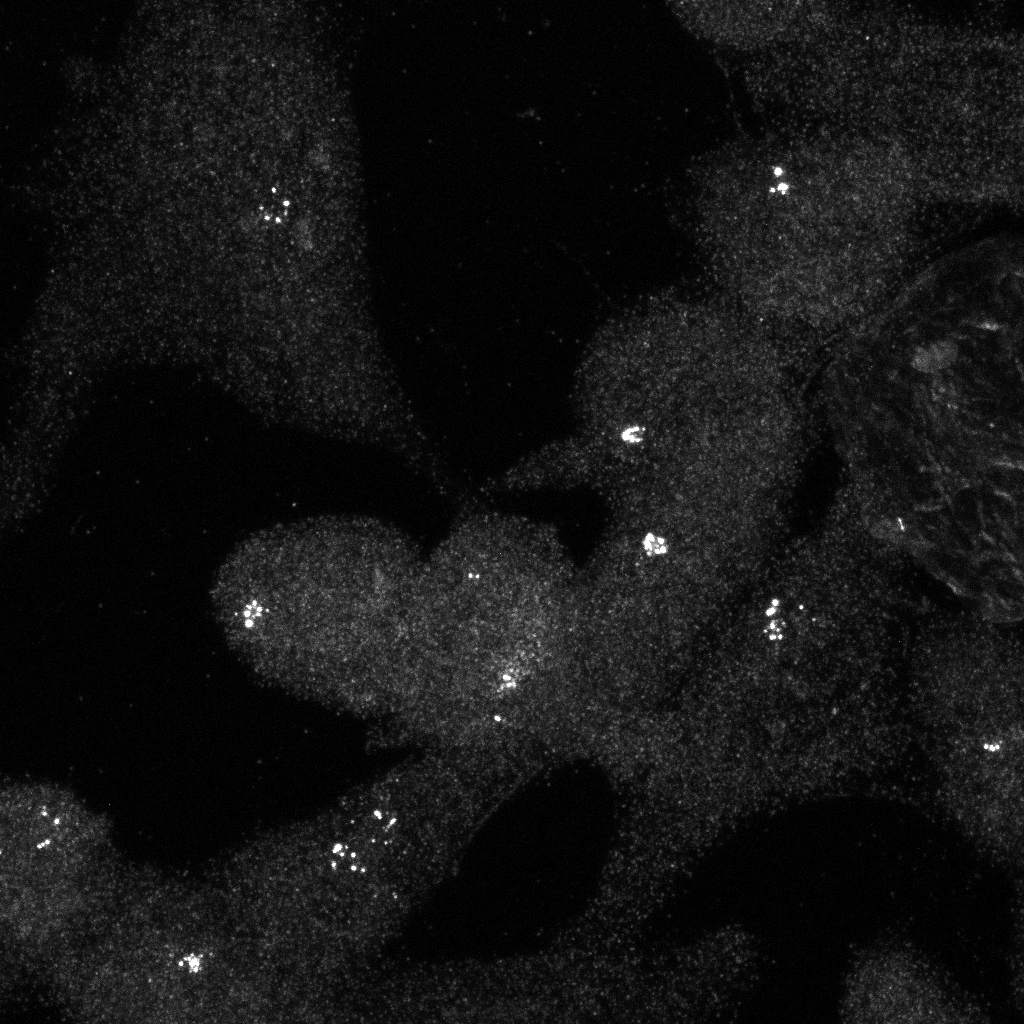

Supplement: Supplementary file 5 — Source data Fig. 2 [file 44319_2026_697_MOESM5_ESM.zip › Figure 2/2C/Dapi_Cep135_CP110_Luciferase siRNA.tif]

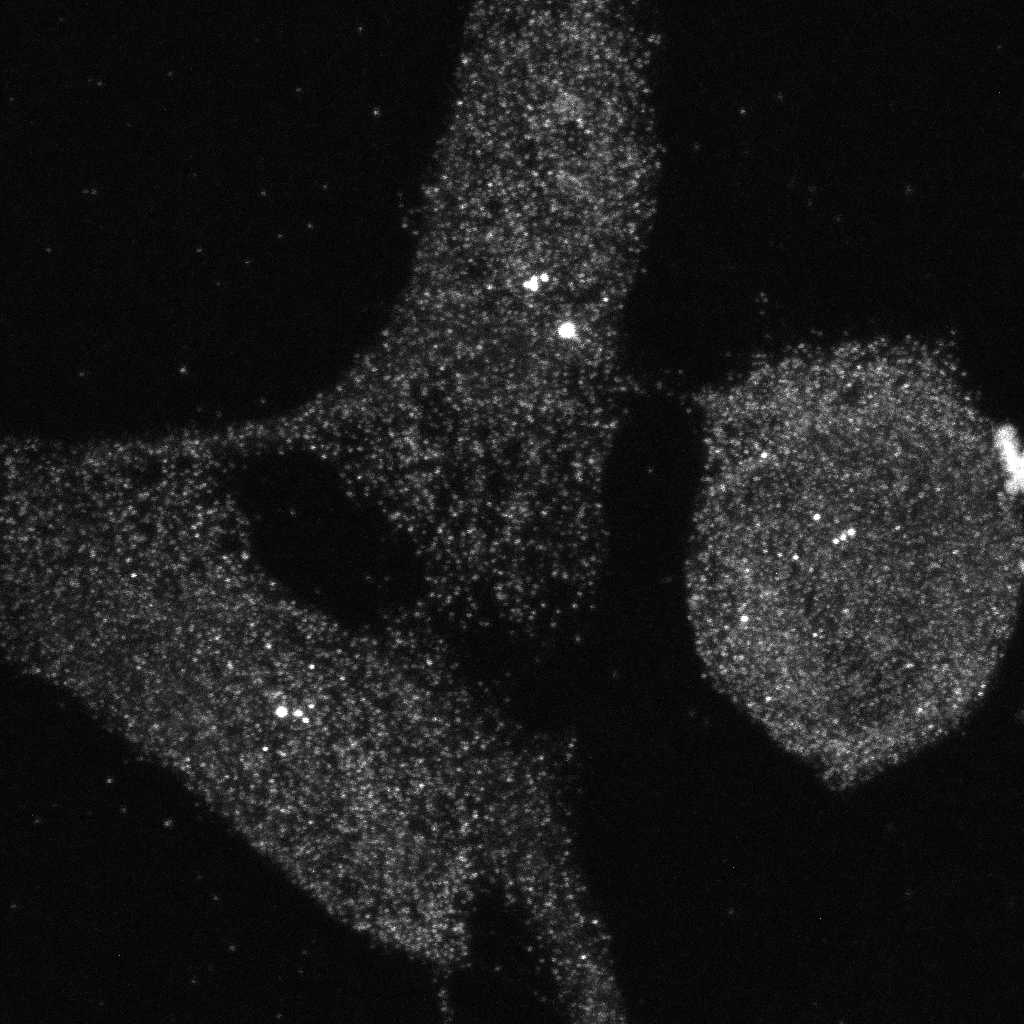

Supplement: Supplementary file 5 — Source data Fig. 2 [file 44319_2026_697_MOESM5_ESM.zip › Figure 2/2C/Dapi_Cep135_CP110_McIdas siRNA.tif]

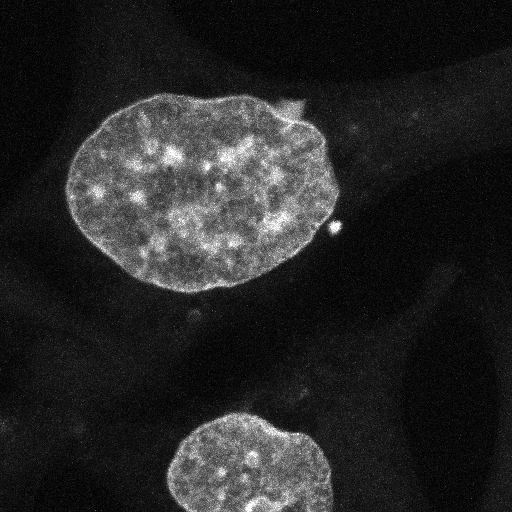

Supplement: Supplementary file 5 — Source data Fig. 2 [file 44319_2026_697_MOESM5_ESM.zip › Figure 2/2E/Dapi_GFP_CP110_Cep135_Luciferase siRNA_GFP.tif]

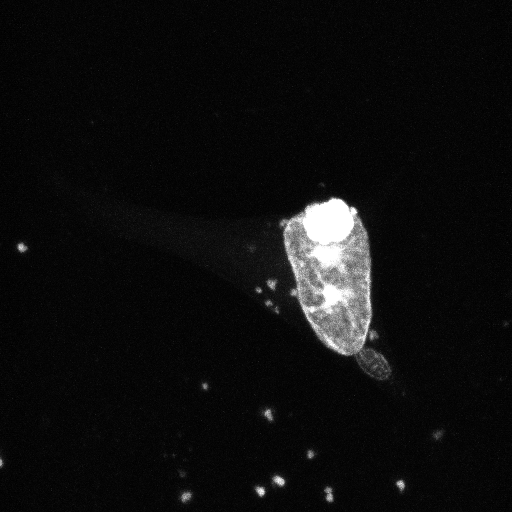

Supplement: Supplementary file 5 — Source data Fig. 2 [file 44319_2026_697_MOESM5_ESM.zip › Figure 2/2E/Dapi_GFP_CP110_Cep135_McIdas siRNA_GFP-McIdas WT.tif]

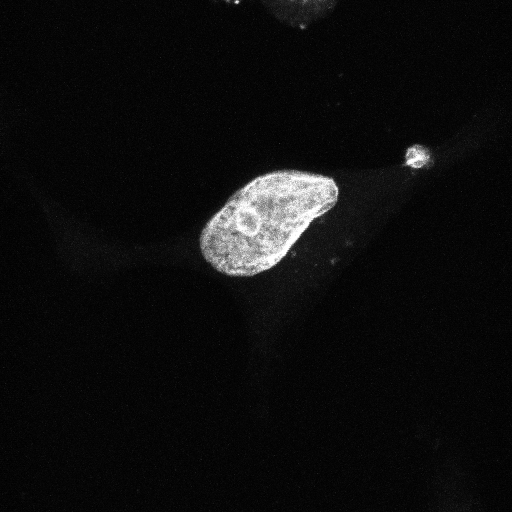

Supplement: Supplementary file 5 — Source data Fig. 2 [file 44319_2026_697_MOESM5_ESM.zip › Figure 2/2E/Dapi_GFP_CP110_Cep135_McIdas siRNA_GFP.tif]

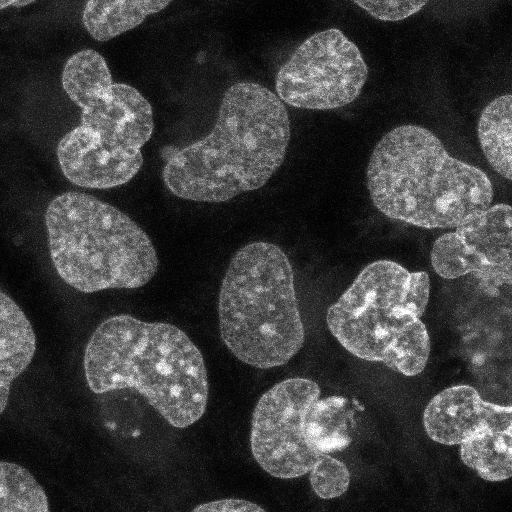

Supplement: Supplementary file 6 — Source data Fig. 3 [file 44319_2026_697_MOESM6_ESM.zip › Figure 3/3C/Dapi_EdU_SAS6_Cep170_Control siRNA.tif]

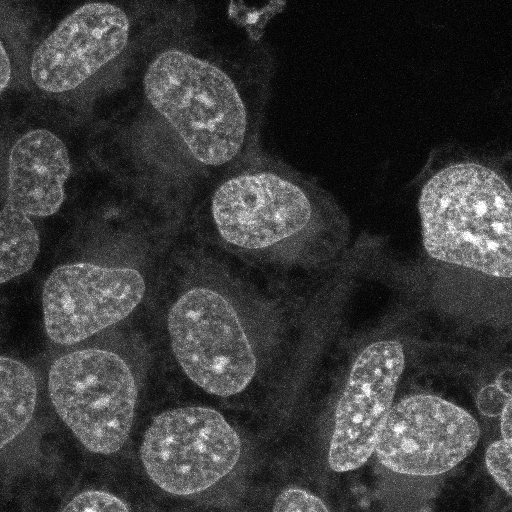

Supplement: Supplementary file 6 — Source data Fig. 3 [file 44319_2026_697_MOESM6_ESM.zip › Figure 3/3C/Dapi_EdU_SAS6_Cep170_McIdas siRNA.tif]

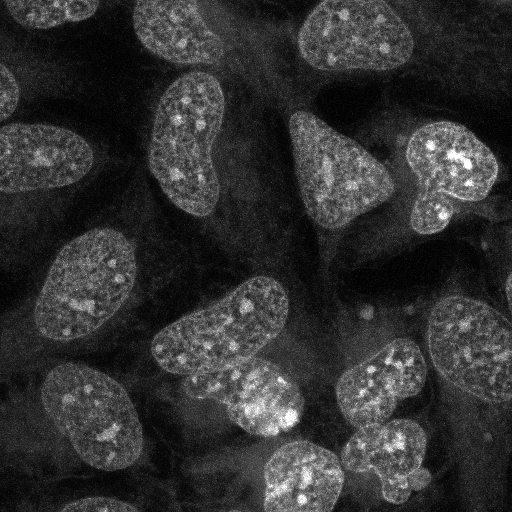

Supplement: Supplementary file 6 — Source data Fig. 3 [file 44319_2026_697_MOESM6_ESM.zip › Figure 3/3A/Dapi_EdU_Centrin_Cep192_McIdas siRNA.tif]

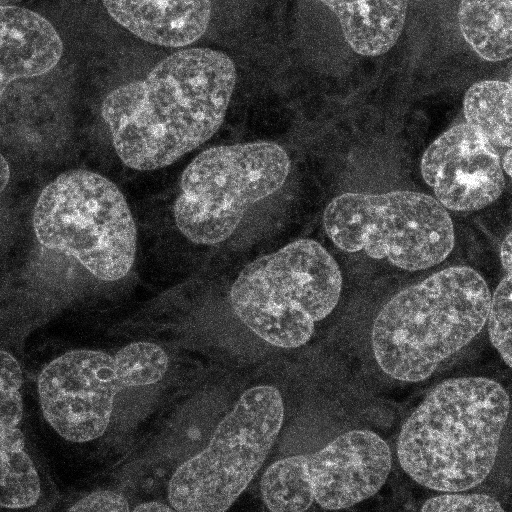

Supplement: Supplementary file 6 — Source data Fig. 3 [file 44319_2026_697_MOESM6_ESM.zip › Figure 3/3A/Dapi_EdU_Centrin_Cep192_Control siRNA.tif]

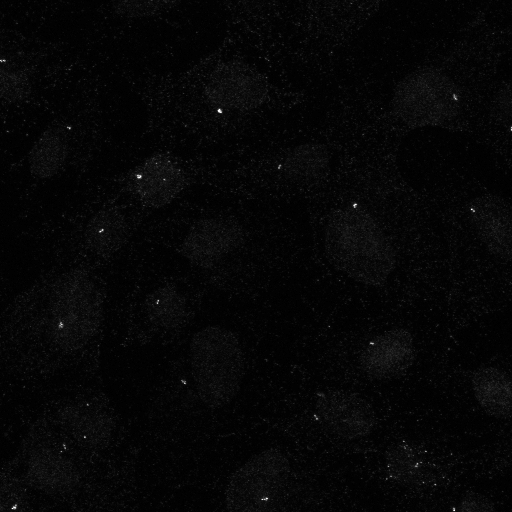

Supplement: Supplementary file 7 — Source data Fig. 4 [file 44319_2026_697_MOESM7_ESM.zip › Figure 4/4C/Centrin_GFP_Dapi_╬│tubulin_GFP-McIdas NES1:2mut.tif]

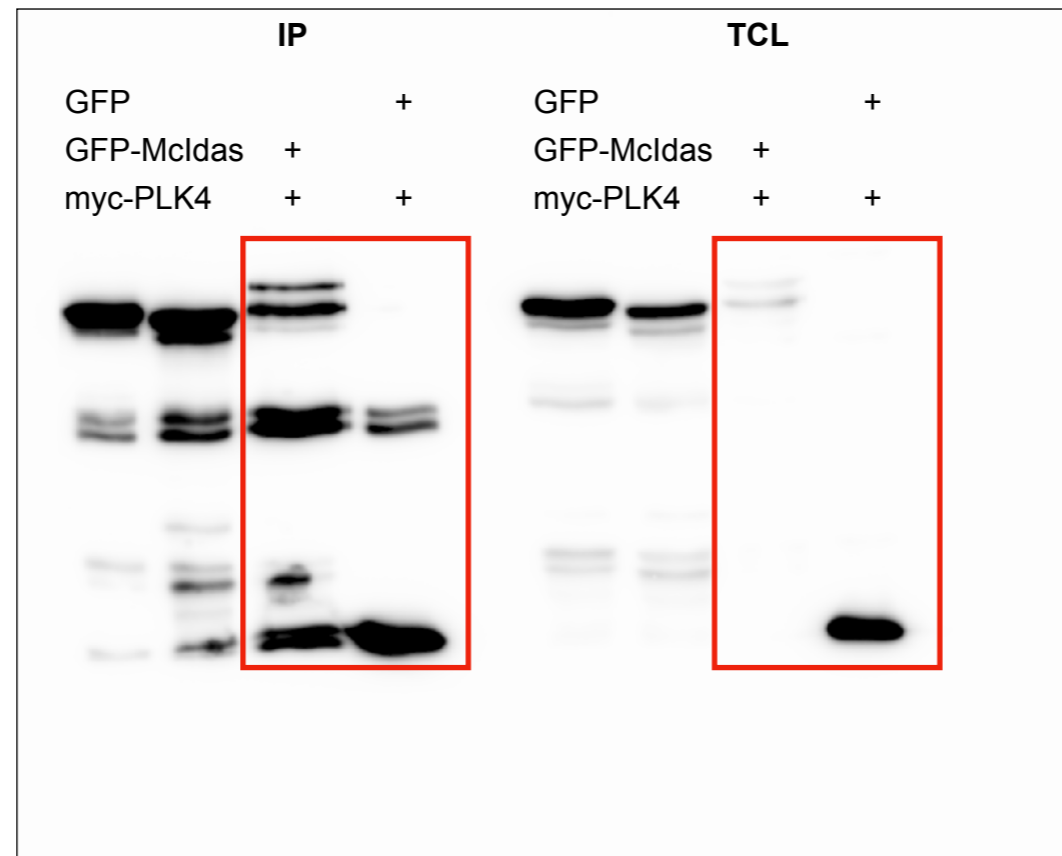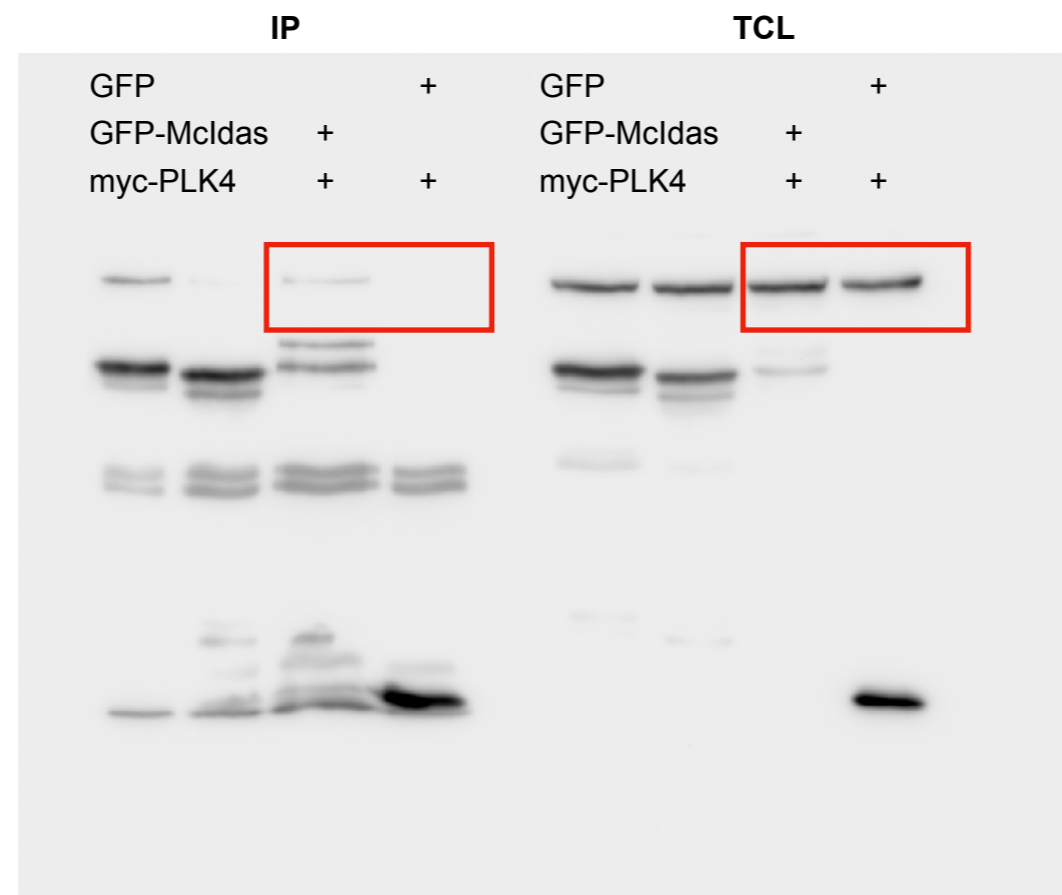

Supplement: Supplementary file 8 — Source data Fig. 5 [file 44319_2026_697_MOESM8_ESM.zip › Figure 5/5A/GFP-McIdas IP_mycPLK4 coIP.pdf]

**<sup>32</sup>P incorporation**

|            |  |   |   |   |   |
|------------|--|---|---|---|---|
| GFP        |  | + | + |   |   |
| GFP-MclDas |  |   |   | + | + |
| PLX4       |  |   | + |   | + |

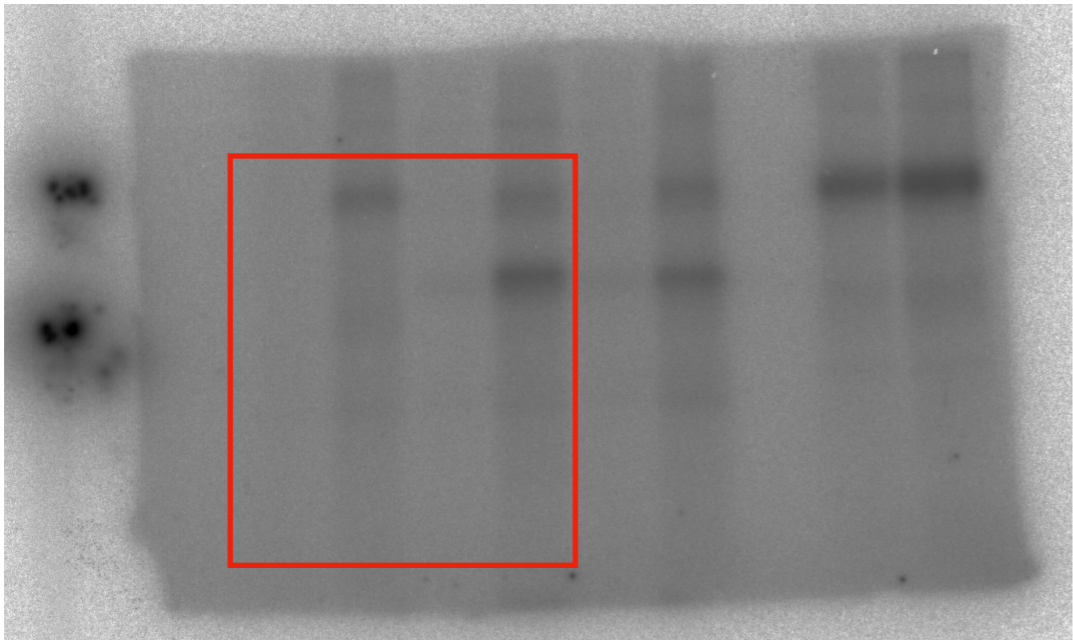

**WB (10% IP)**

|            |  |   |   |   |   |
|------------|--|---|---|---|---|
| GFP        |  |   |   | + | + |
| GFP-MclDas |  | + | + |   |   |
| PLX4       |  | + |   | + |   |

anti-GFP

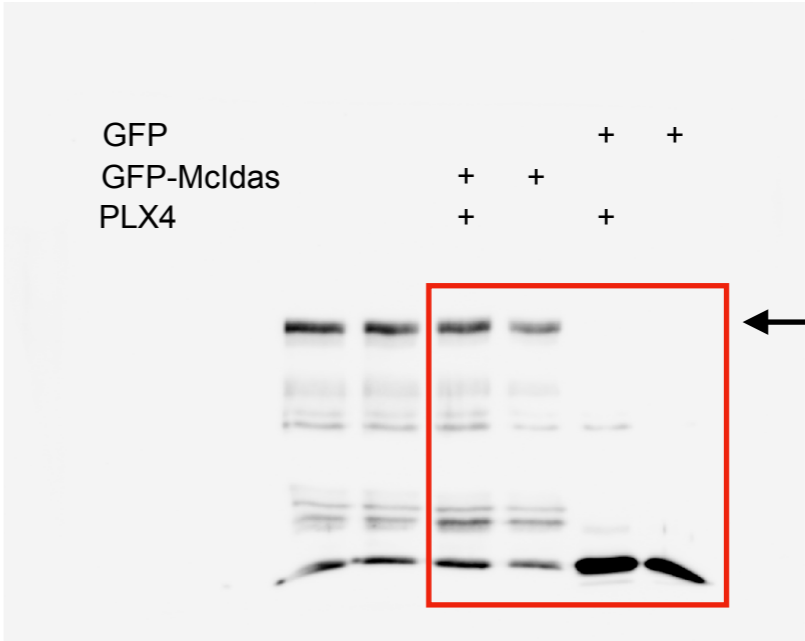

MclDas

anti-PLK4

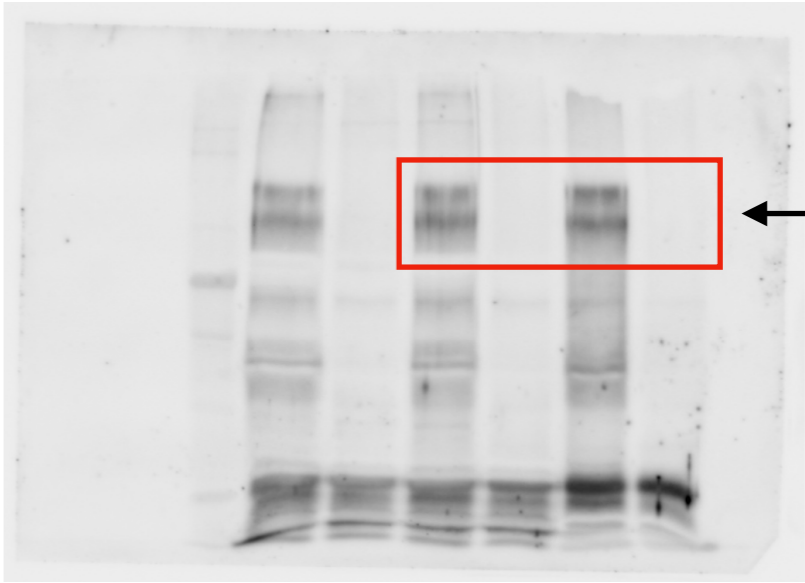

PLK4

Supplement: Supplementary file 8 — Source data Fig. 5 [file 44319_2026_697_MOESM8_ESM.zip › Figure 5/5C/In vitro phosphorylation assay_WB 10%IP.pdf]

**Input**

GFP  
GFP-Mcldas

+ +

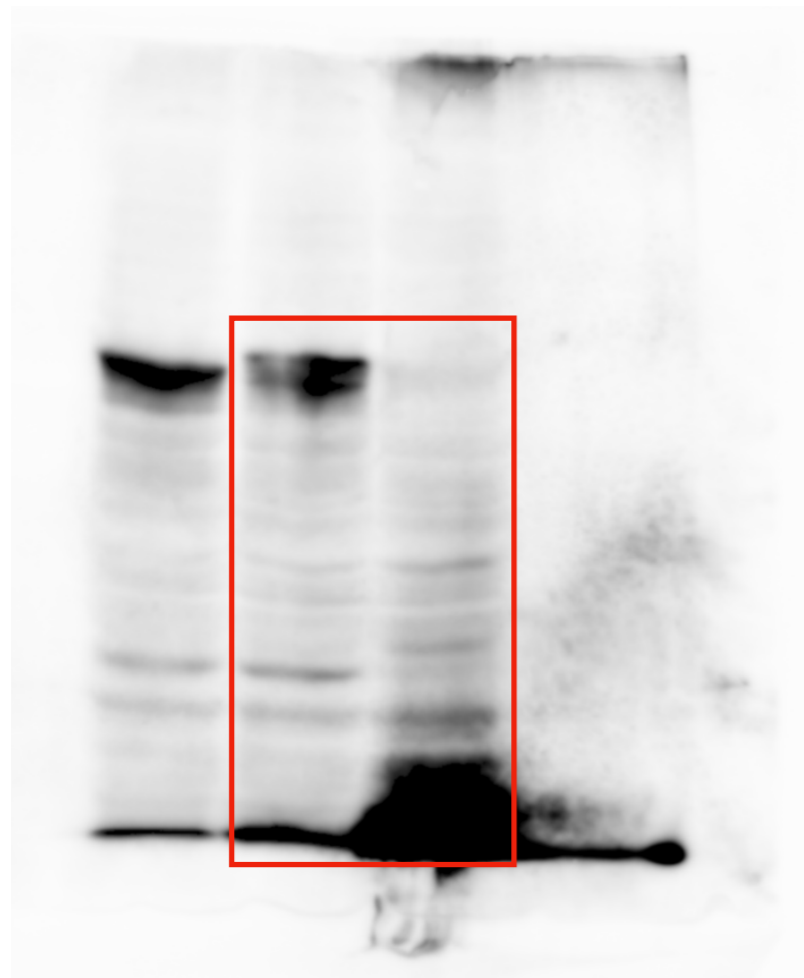

anti-GFP

GFP  
GFP-Mcldas +

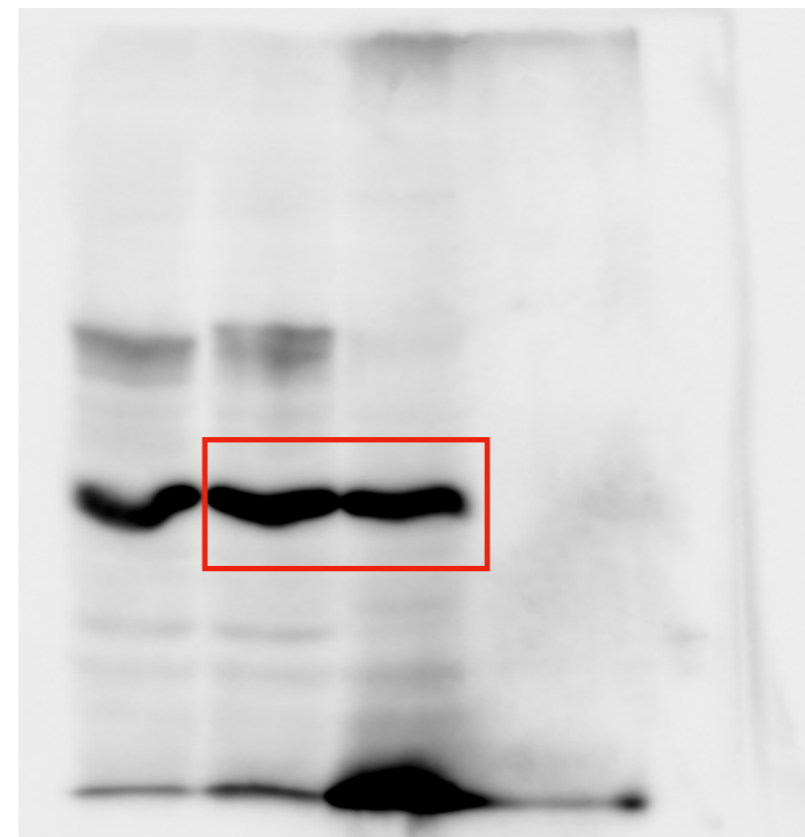

anti-Tubulin

Supplement: Supplementary file 8 — Source data Fig. 5 [file 44319_2026_697_MOESM8_ESM.zip › Figure 5/5D/Input.pdf]

|                      |   |   |   |   |   |   |   |
|----------------------|---|---|---|---|---|---|---|
| Mcdas 1-179          | + | + |   |   |   |   |   |
| Mcdas 101-284        |   |   | + | + |   |   |   |
| Mcdas 314-385        |   |   |   |   | + | + |   |
| Non-specific protein |   |   |   |   |   |   | + |
| PLX4                 |   | + |   | + |   | + | + |

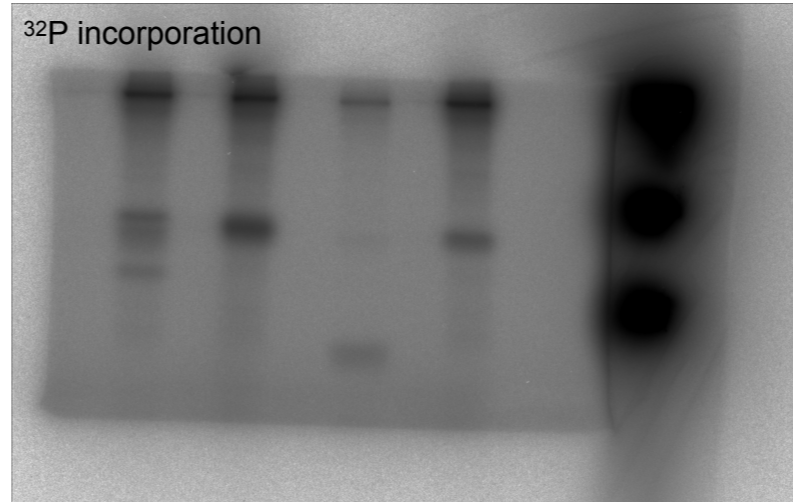

|                      |   |   |   |   |   |   |   |
|----------------------|---|---|---|---|---|---|---|
| Mcdas 1-179          | + | + |   |   |   |   |   |
| Mcdas 101-284        |   |   | + | + |   |   |   |
| Mcdas 314-385        |   |   |   |   | + | + |   |
| Non-specific protein |   |   |   |   |   |   | + |
| PLX4                 |   | + |   | + |   | + | + |

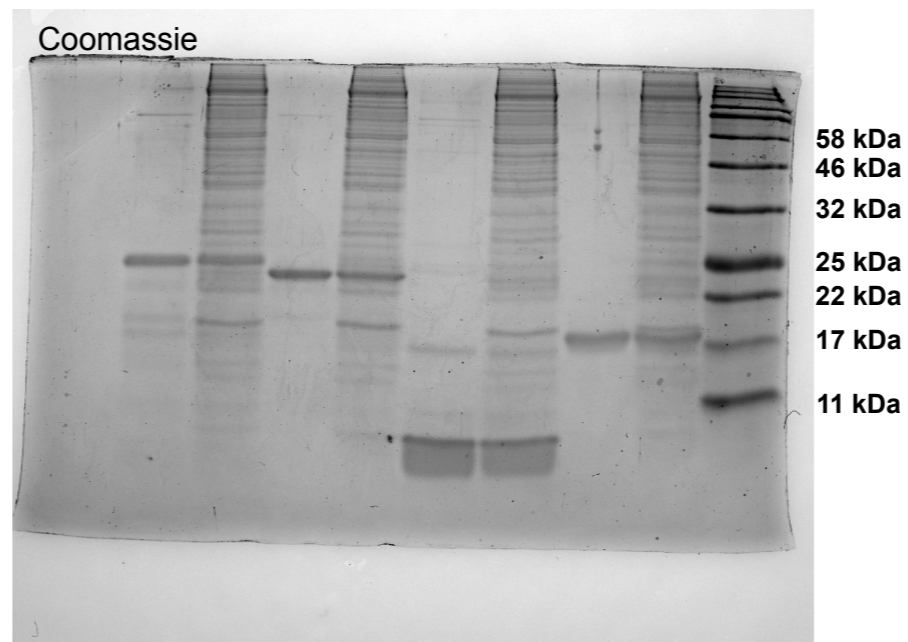

Supplement: Supplementary file 8 — Source data Fig. 5 [file 44319_2026_697_MOESM8_ESM.zip › Figure 5/5E/In vitro phosphorylation assay_recombinant proteins.pdf]

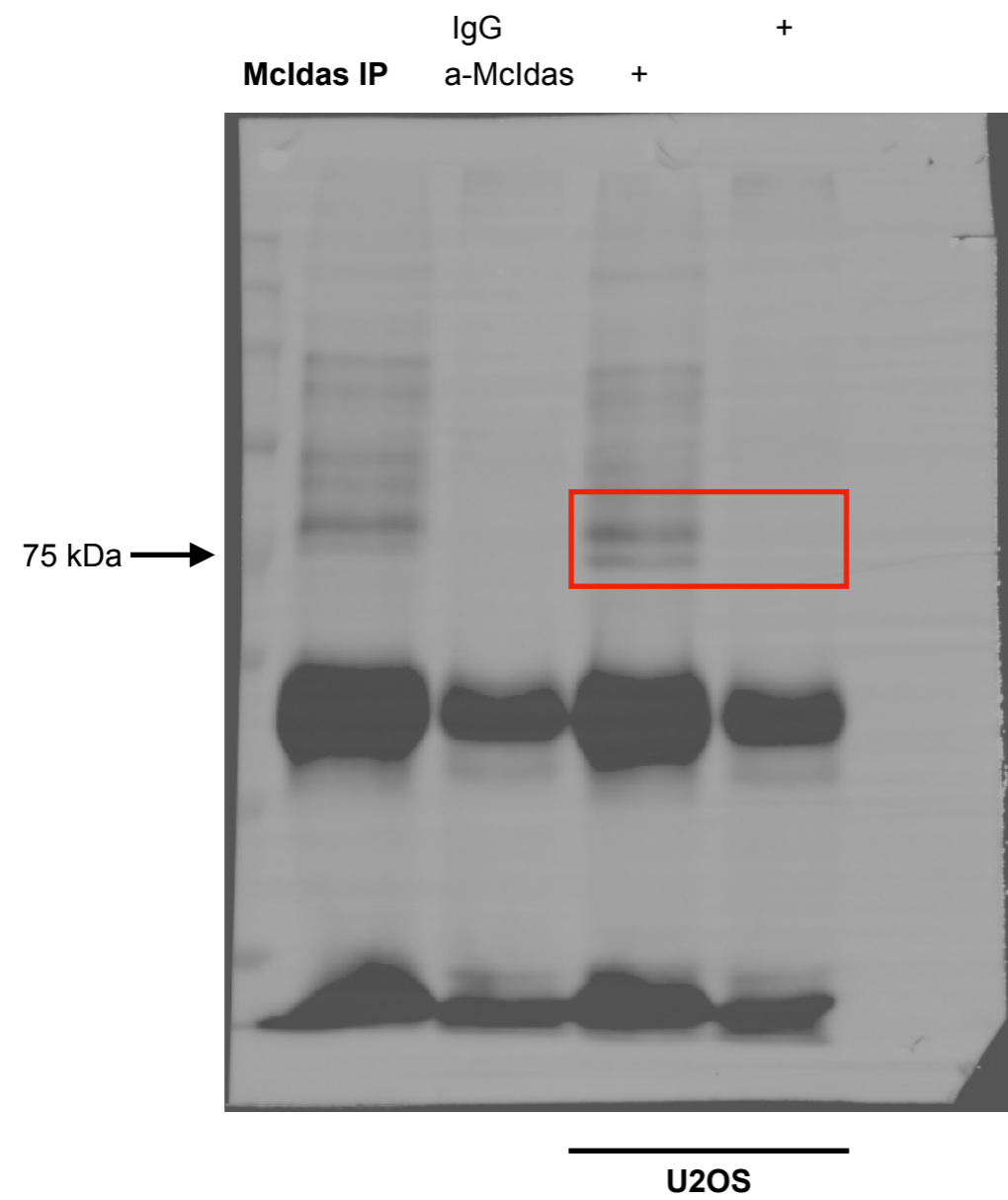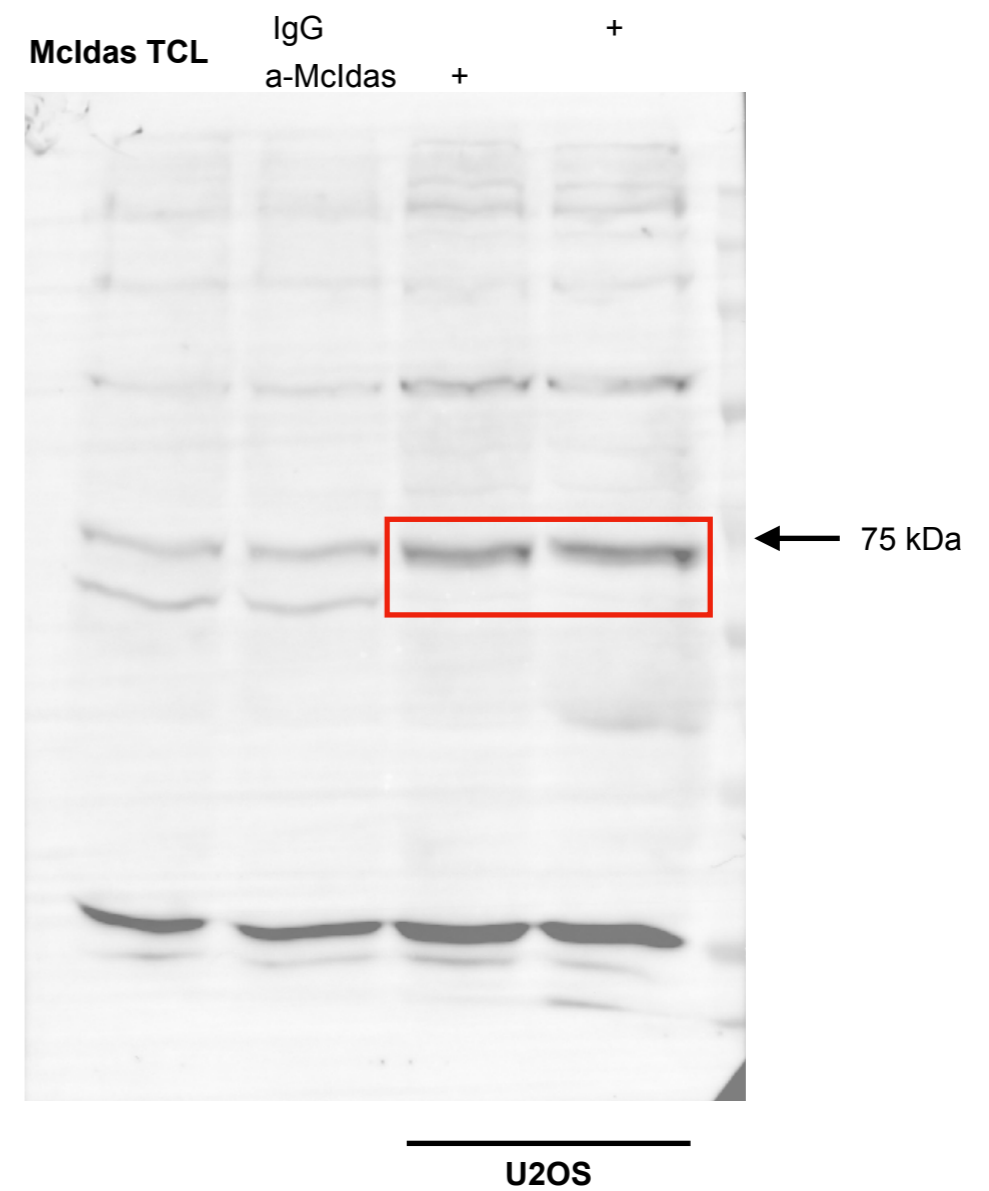

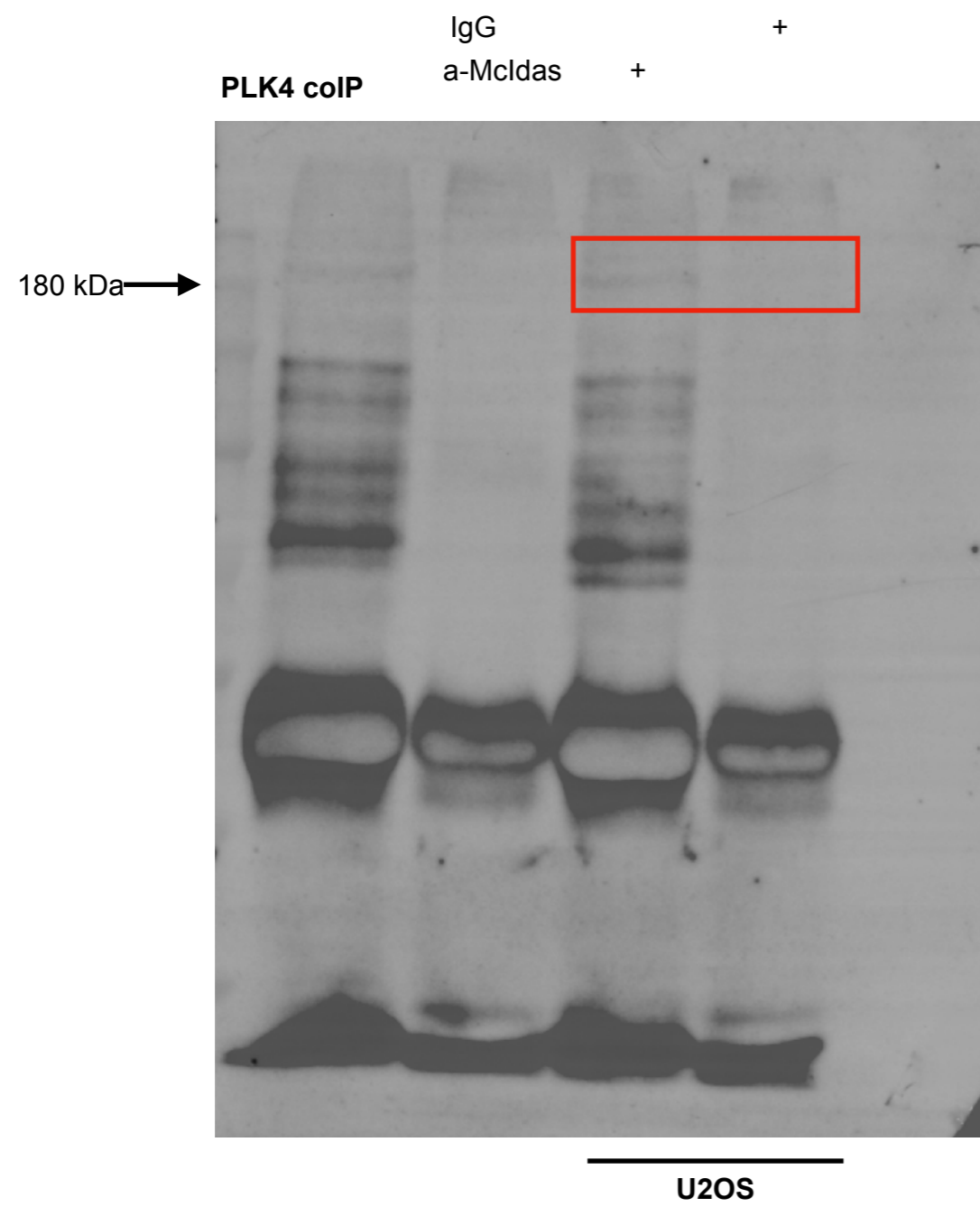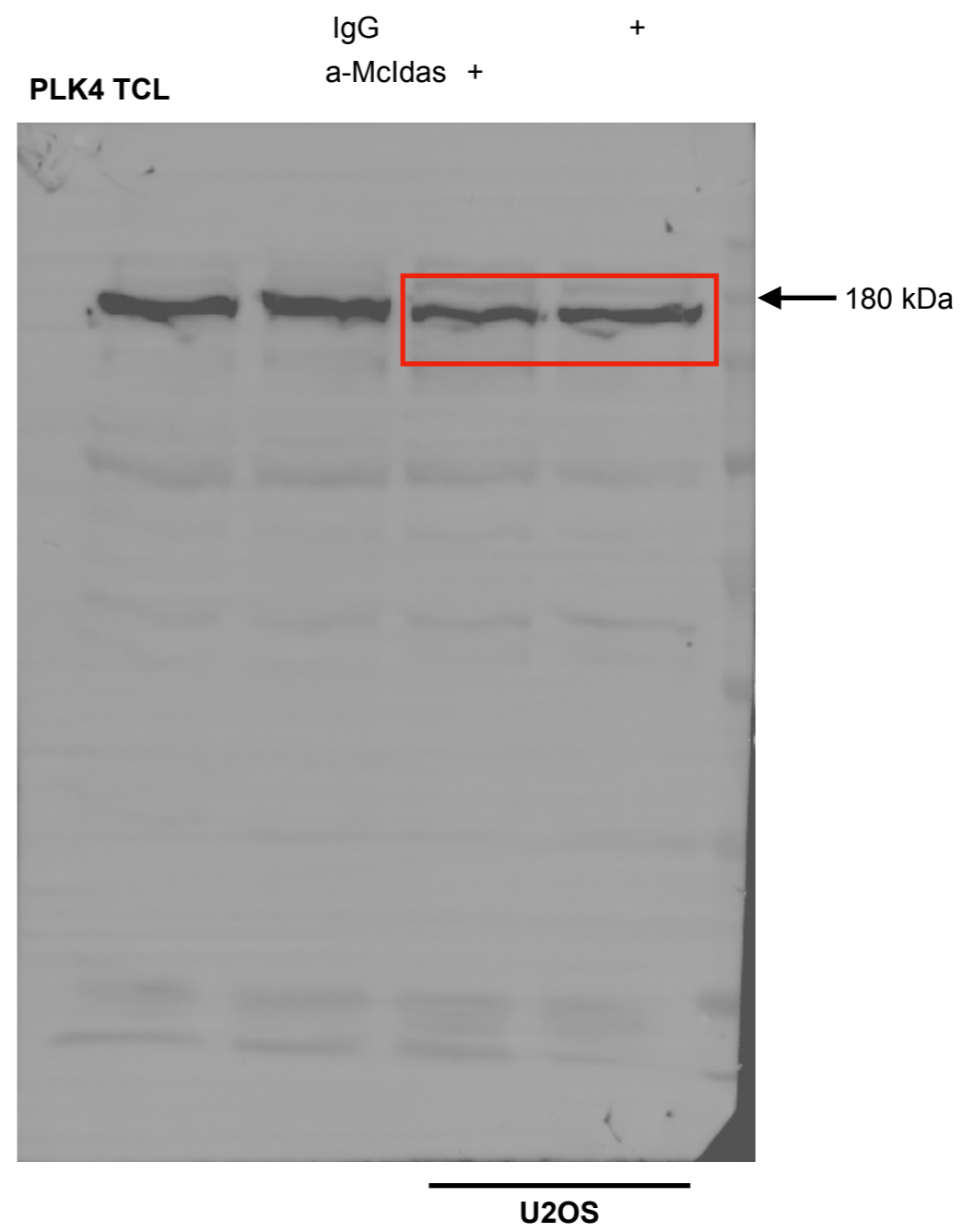

Supplement: Supplementary file 8 — Source data Fig. 5 [file 44319_2026_697_MOESM8_ESM.zip › Figure 5/5B/Idas_PLK4_IPs_endogenous proteins.pdf]

GFP-MclDas

WT

10A

10D

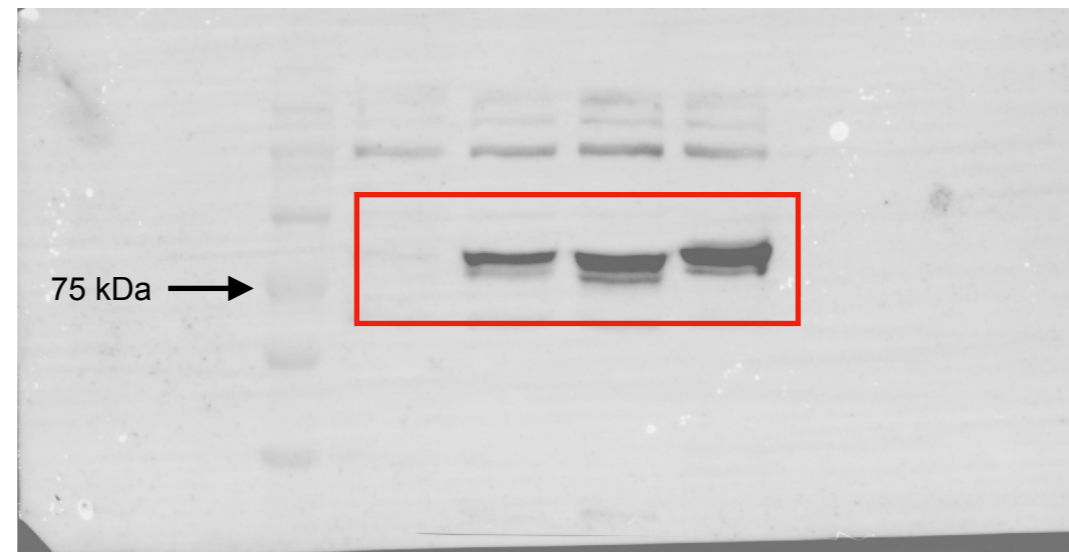

anti-GFP

GFP

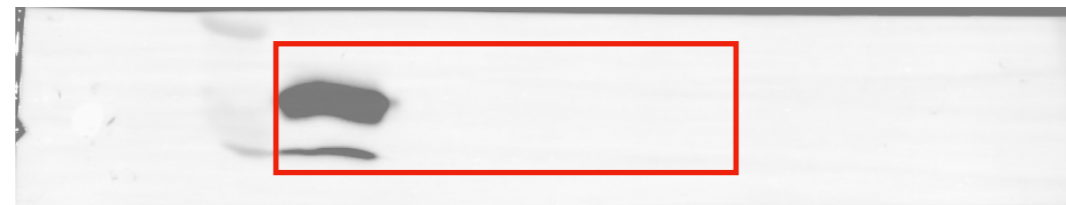

anti-GFP

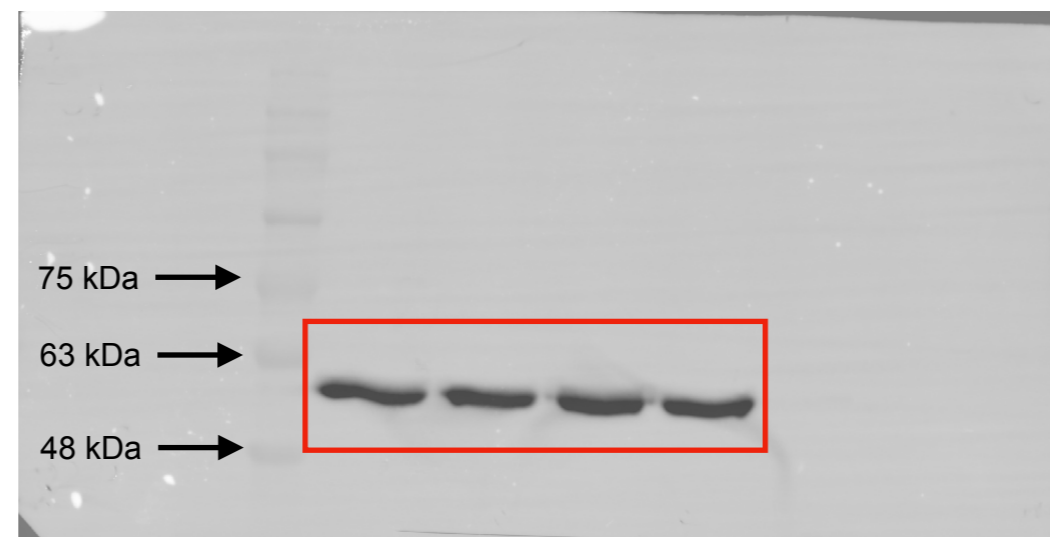

anti-Tubulin

Supplement: Supplementary file 10 — Source data Fig. 7 [file 44319_2026_697_MOESM10_ESM.zip › Figure 7/7C/WB_McIdas_Tubulin.pdf]

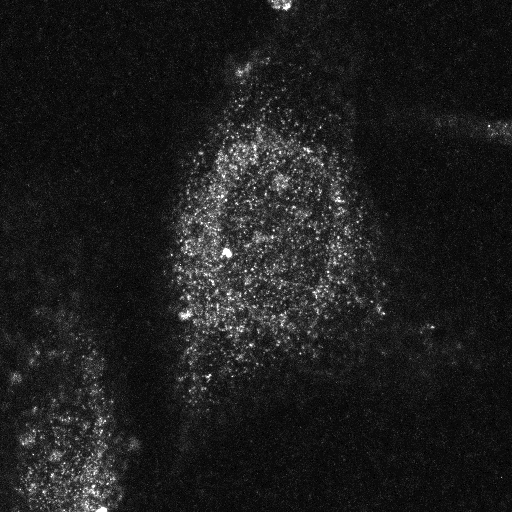

Supplement: Supplementary file 10 — Source data Fig. 7 [file 44319_2026_697_MOESM10_ESM.zip › Figure 7/7A/Dapi_GFP_Cep135_CP110_GFP.tif]

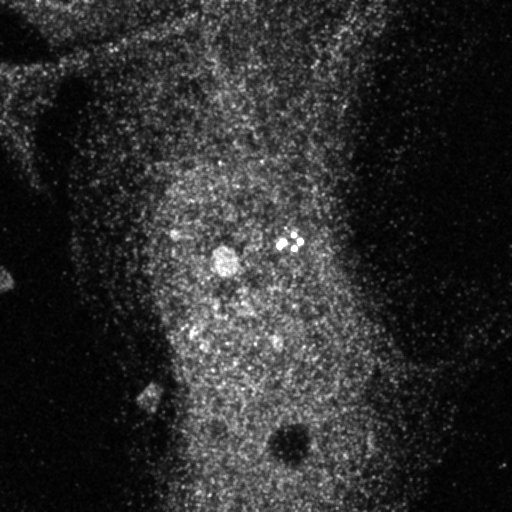

Supplement: Supplementary file 10 — Source data Fig. 7 [file 44319_2026_697_MOESM10_ESM.zip › Figure 7/7A/Dapi_GFP_Cep135_CP110_GFP-McIdas WT.tif]

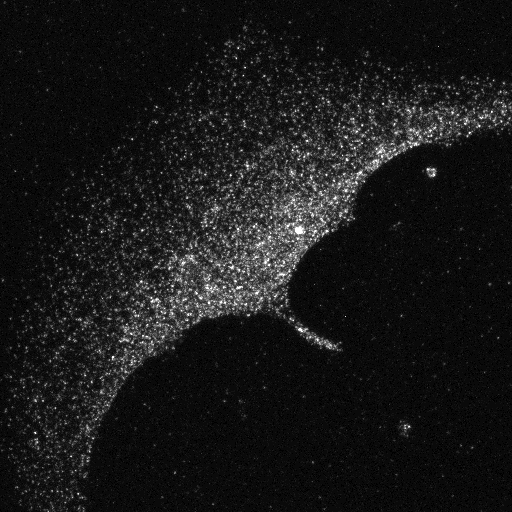

Supplement: Supplementary file 10 — Source data Fig. 7 [file 44319_2026_697_MOESM10_ESM.zip › Figure 7/7A/Dapi_GFP_Cep135_CP110_GFP-McIdas 10D.tif]

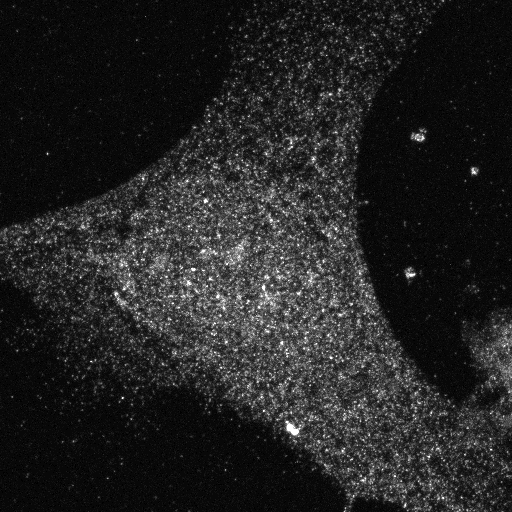

Supplement: Supplementary file 10 — Source data Fig. 7 [file 44319_2026_697_MOESM10_ESM.zip › Figure 7/7A/Dapi_GFP_Cep135_CP110_GFP-McIdas 10A.tif]
