# Supplementary material for: McIdas localizes to centrioles and controls centriole numbers through PLK4-dependent phosphorylation
Source: EMBO Rep. 2026 Feb 5;27(6):1478–509. doi: 10.1038/s44319-026-00697-5 (PMC13022133; doi:10.1038/s44319-026-00697-5)
Supplement: Supplementary file 9 — Source data Fig. 6 [file 44319_2026_697_MOESM9_ESM.zip › Figure 6/6C/In vitro phospho assay_WB 20% IP.pdf]

^{32}P incorporation

GFP	+		
GFP-McIdas WT		+	
GFP-McIdas 10A			+
PLX4	+	+	+

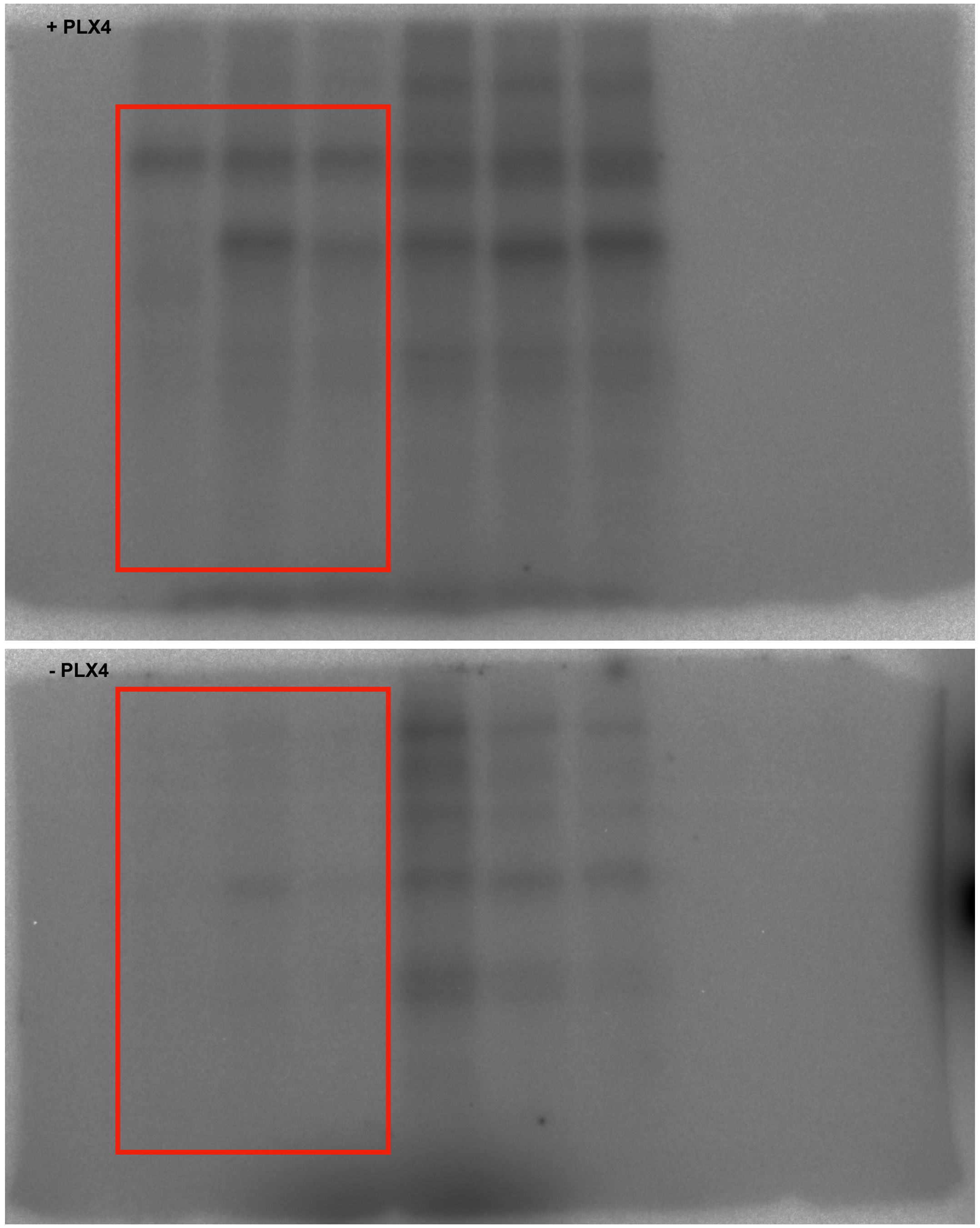

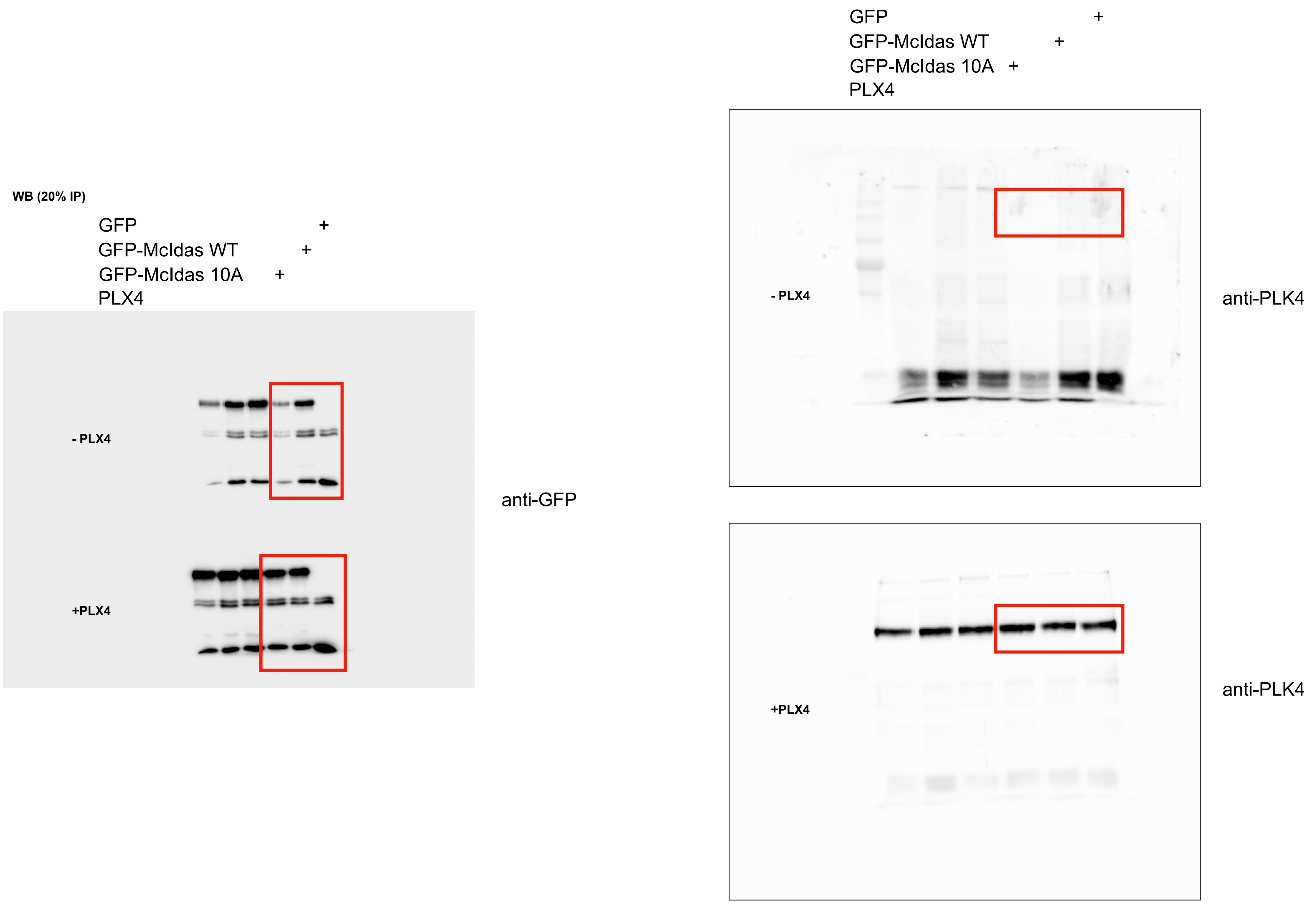

WB (20% IP)
GFP +
GFP-McIdas WT +
GFP-McIdas 10A +
PLX4
- PLX4
+PLX4
anti-GFP
GFP +
GFP-McIdas WT +
GFP-McIdas 10A +
PLX4
- PLX4
anti-PLK4
+PLX4
anti-PLK4
