# Supplementary material for: McIdas localizes to centrioles and controls centriole numbers through PLK4-dependent phosphorylation
Source: EMBO Rep. 2026 Feb 5;27(6):1478–509. doi: 10.1038/s44319-026-00697-5 (PMC13022133; doi:10.1038/s44319-026-00697-5)
Supplement: Supplementary file 11 — Expanded View Figures [file 44319_2026_697_MOESM11_ESM.pdf]

Expanded View Figures

Figure EV1. McIdas localizes to centrosomes.

(**A**) U2OS cells were immunostained with antibodies against McIdas and Centrin (a distal lumen centriole marker). G1-phase cells were identified as EdU-negative and exhibiting two Centrin dots. (**B**) Quantification of G1-phase centrosomes showing either one (light green) or two (dark green) McIdas dots at centrosomes. Data are from two independent experiments and at least 20 G1-phase centrosomes counted in each experiment. Error bars indicate ± SEM and *P*-values were calculated using a two-tailed Student's t-test: ns, not significant (*P* value: $P = 0.2020$). (**C**) U2OS and hTERT RPE-1 cells were transfected with vectors expressing either GFP-tagged McIdas or GFP alone as a control. Cells were pre-extracted and immunostained with antibodies against GFP (to mark transfected cells), McIdas and Centrin. Arrows indicate centrosomes; asterisks show Centrin accumulation observed upon GFP-McIdas overexpression. Maximum intensity projection images are shown. Protein aggregates observed in GFP-McIdas overexpressing cells are not considered specific. (**D**) HeLa cells stably expressing GFP-tagged McIdas or GFP alone were treated with the proteasome inhibitor MG132 for 4 h, fixed and immunostained with antibodies against GFP, endogenous McIdas and Cep135 (a proximal centriole marker). (**E**) Representative image of a prophase hTERT RPE-1 cell showing duplicated centrosomes stained with antibodies against McIdas, Centrin and Cep164. (**F**, **G**) Corresponding fluorescence intensity plots of the centrosomal signals shown in (**E**) for McIdas, Cep164 and Centrin. The position and direction of the line used to generate the intensity profiles as a function of distance are indicated in (**E**) by the white line and arrow, respectively. Schematic representation in (**E**) shows McIdas localization relative to the centriole markers, consistent with the intensity profile analysis. White boxes indicate regions shown as higher-magnification images. DNA was stained with Hoechst. Scale bars, 5 µm. ns not significant, AU arbitrary units.

 © The Author(s)

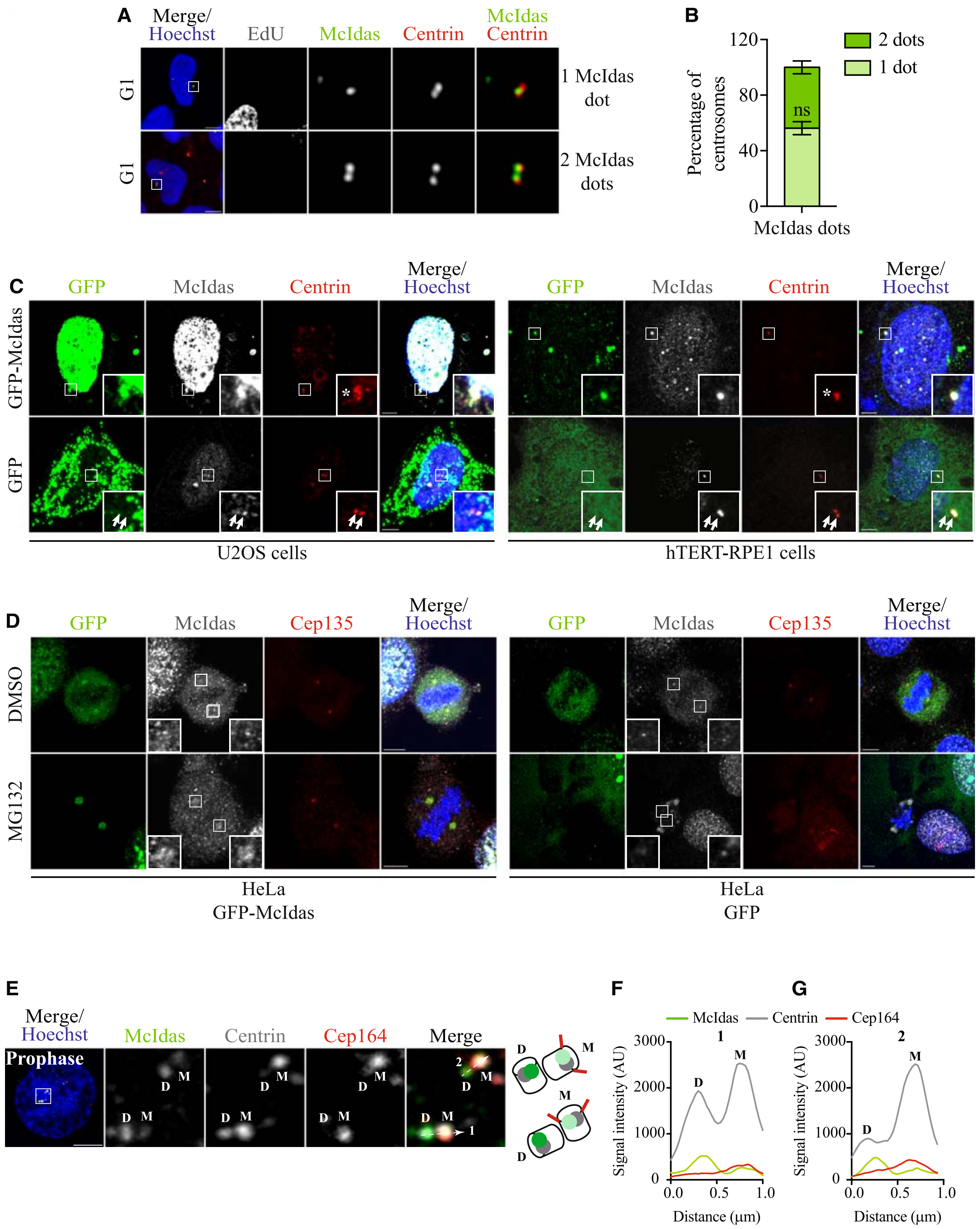
A
Merge/ Hoechst
EdU
McIdas
Centrin
McIdas Centrin
G1
G1
1 McIdas dot
2 McIdas dots
B
Percentage of centrosomes
2 dots
1 dot
ns
McIdas dots
C
GFP
McIdas
Centrin
Merge/ Hoechst
GFP-McIdas
GFP
U2OS cells
hTERT-RPE1 cells
D
GFP
McIdas
Cep135
Merge/ Hoechst
DMSO
MG132
HeLa GFP-McIdas
HeLa GFP
E
Merge/ Hoechst
McIdas
Centrin
Cep164
Merge
Prophase
F
G
McIdas
Centrin
Cep164
Signal intensity (AU)
Distance (µm)

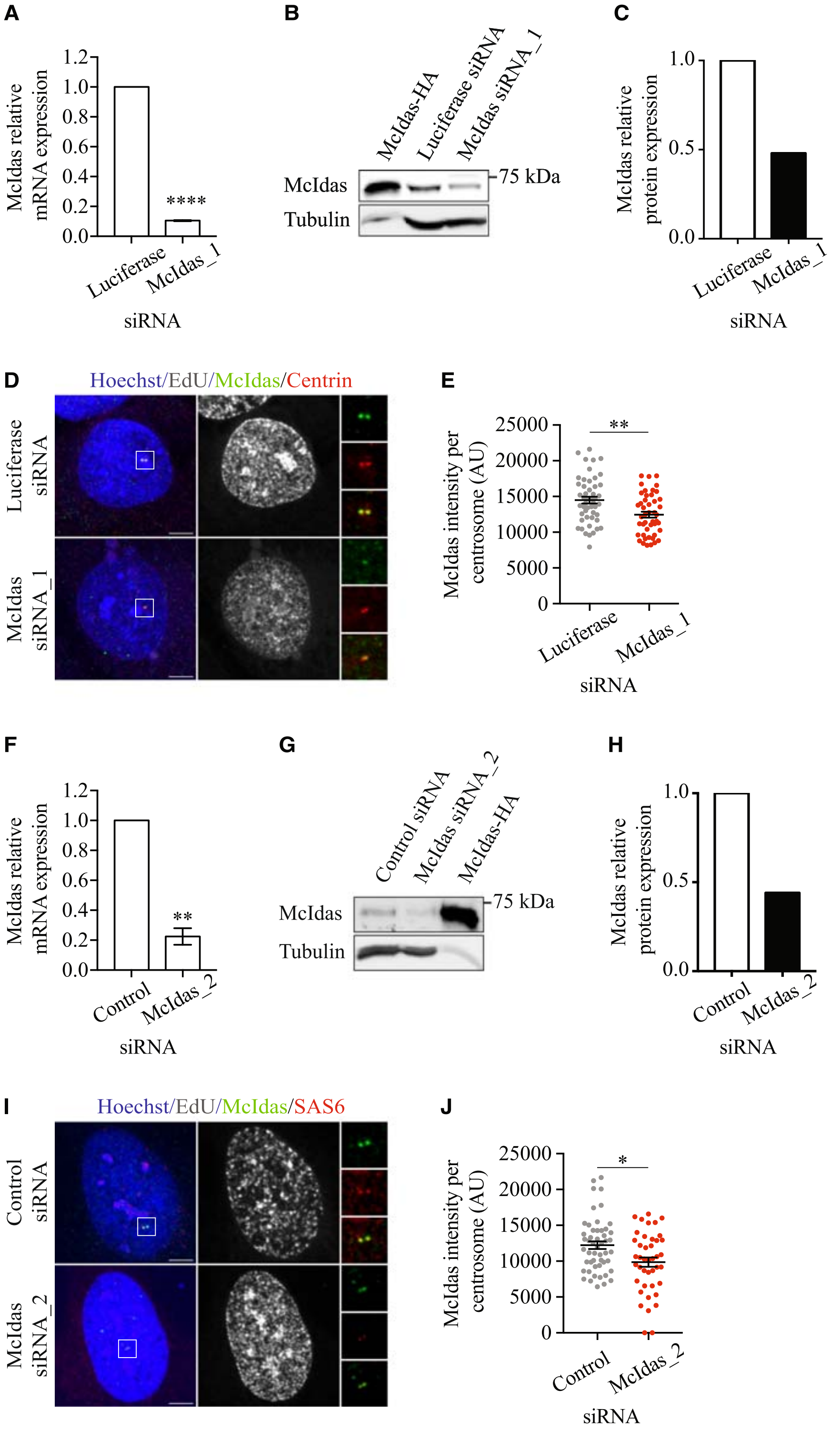
A
McIdas relative mRNA expression
1.2
1.0
0.8
0.6
0.4
0.2
0.0
****
Luciferase
McIdas_1
siRNA
B
McIdas-HA
Luciferase siRNA
McIdas siRNA_1
McIdas
Tubulin
75 kDa
C
McIdas relative protein expression
1.0
0.5
0.0
Luciferase
McIdas_1
siRNA
D
Hoechst/EdU/McIdas/Centrin
Luciferase siRNA
McIdas siRNA_1
E
McIdas intensity per centrosome (AU)
25000
20000
15000
10000
5000
0
**
Luciferase
McIdas_1
siRNA
F
McIdas relative mRNA expression
1.2
1.0
0.8
0.6
0.4
0.2
0.0
**
Control
McIdas_2
siRNA
G
Control siRNA
McIdas siRNA_2
McIdas-HA
McIdas
Tubulin
75 kDa
H
McIdas relative protein expression
1.0
0.5
0.0
Control
McIdas_2
siRNA
I
Hoechst/EdU/McIdas/SAS6
Control siRNA
McIdas siRNA_2
J
McIdas intensity per centrosome (AU)
25000
20000
15000
10000
5000
0
*
Control
McIdas_2
siRNA

 © The Author(s)

Figure EV2. Verification of McIdas localization to centrosomes.

Two different McIdas siRNA oligos (1 and 2) were used to verify the specificity of McIdas localization to centrosomes. U2OS cells were transfected with luciferase or McIdas siRNA_1 (**A–E**) and control or McIdas siRNA_2 (**F–J**). Cells were collected and McIdas mRNA (**A**, **F**) and protein levels (**B**, **C** and **G**, **H**) were assessed by qPCR and western blot analysis, respectively. Cells were fixed and immunostained with antibodies against McIdas (green) and Centrin (red, a distal lumen centriole marker) (**D**) or with antibodies against McIdas (green) and SAS6 (red, a centriole marker) (**I**). McIdas fluorescence intensity at centrosomes (**E**, **J**) was quantified. For the quantifications of McIdas centrosomal levels at least 40 centrosomes were counted per condition. Two independent experiments were conducted for all data shown. Error bars indicate ± SEM. *P*-values in (**A**) ($P < 0.0001$) & (**F**) ($P = 0.0068$) were calculated using two-tailed Student's t- and *P*-values in (**E**) ($P = 0.0034$) and (**J**) ($P = 0.0274$) were calculated by the nonparametric two-tailed Mann-Whitney test: *$P < 0.1$, **$P < 0.01$, ****$P < 0.0001$. White boxes indicate regions shown as higher-magnification images. DNA was stained with Hoechst. Scale bars, 5 µm. AU arbitrary units.

© The Author(s)

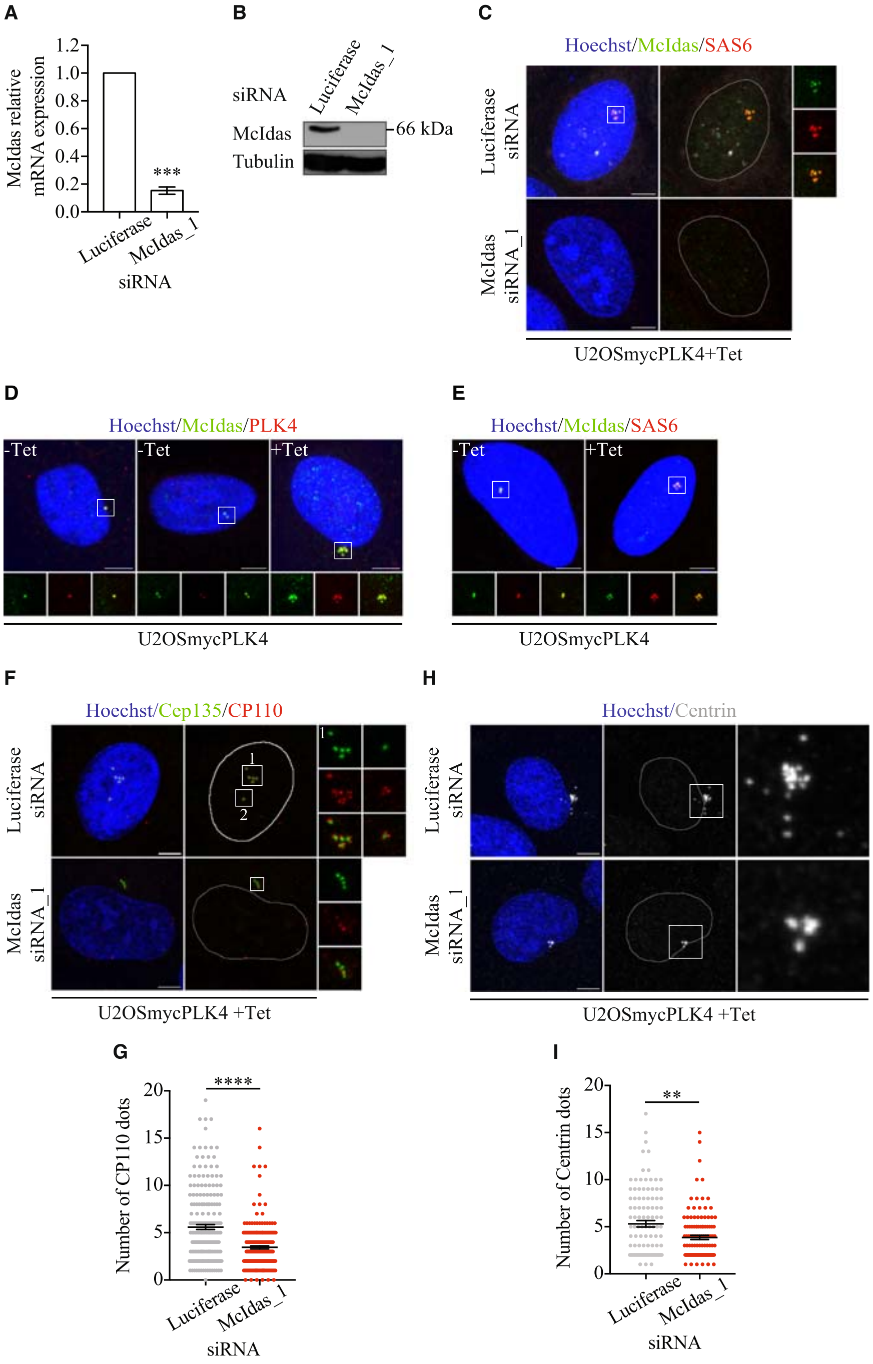

Figure EV3. Mcldas is required for PLK4-induced centriole amplification.

(**A, B**) U2OS cells expressing myc-PLK4 under a tetracycline-dependent promoter were transfected with Mcldas_1 or control siRNA oligos. A second transfection was performed 24 h later, followed by tetracycline treatment to induce myc-PLK4 overexpression. Mcldas mRNA (**A**) and protein (**B**) levels were assessed by qPCR and western blot, respectively. *P*-value ($P = 0.0005$) was calculated using two-tailed Student's t-test: ***$P < 0.001$. (**C**) Cells were fixed and immunostained with antibodies against Mcldas and SAS6. (**D, E**) U2OS cells expressing myc-PLK4 under a tetracycline-dependent promoter were treated with tetracycline or DMSO as a control and immunostained with antibodies against Mcldas and PLK4 (**D**) or SAS6 (**E**). (**F–I**) U2OS cells expressing myc-PLK4 under a tetracycline-dependent promoter were transfected with Mcldas_1 or control siRNA oligos. A second siRNA transfection was performed 24 h later followed by tetracycline treatment to induce myc-PLK4 overexpression. Cells were fixed and immunostained for CP110 and Cep135 (**F**) or Centrin (**H**) to count centriole numbers. (**G, I**) Quantification of centriole numbers in control and Mcldas-depleted cells overexpressing PLK4. The number of CP110 or Centrin dots corresponds to the number of centrioles. Data are from two independent experiments. In each experiment, more than 100 cells were counted per condition. Error bars indicate ± SEM. *P*-values in (**G**) ($P < 0.0001$) and (**I**) ($P = 0.0025$) were calculated using the nonparametric two-tailed Mann-Whitney test: **P <0.01, ****P <0.0001. Nuclear boundaries are outlined and white boxes indicate regions shown as higher-magnification images. DNA was stained with Hoechst. Scale bars, 5 μm. Tet tetracycline.

 © The Author(s)

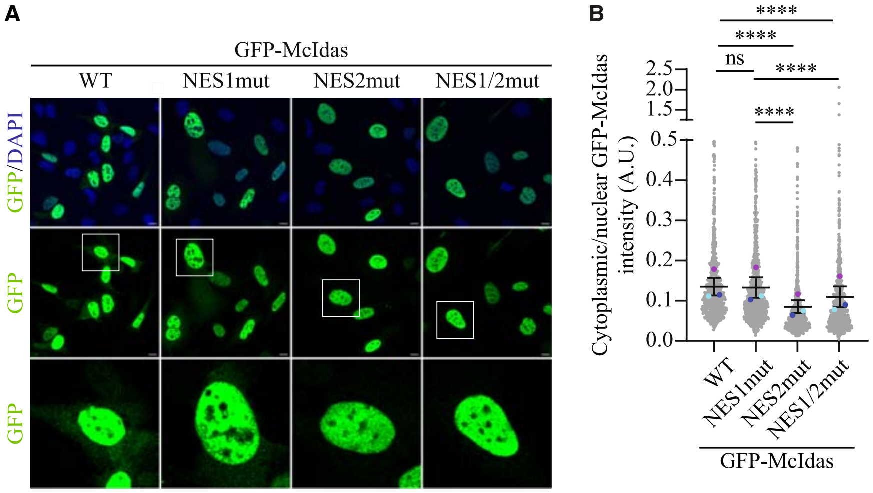

Figure EV4. McIdas cytoplasmic localization is mediated by a nuclear export signal.

(**A**) U2OS cells were transfected with vectors expressing GFP-McIdas WT or the GFP-McIdas NES mutants. After 48 h, cells were fixed and stained for GFP. (**B**) Quantification of the cytoplasmic to nuclear GFP signal intensity ratio for each condition. Small symbols represent individual cells and large symbols represent the mean values of three independent experiments. Error bars represent ± SEM. At least 100 cells were counted per condition in each experiment. *P*-values were calculated using two-tailed Student's t-test: ****$P < 0.0001$ and ns, not significant (*P* values: WT-NES1mut, $P = 0.9811$; WT-NES2mut, $P < 0.0001$; WT-NES1/2mut, $P < 0.0001$; NES1mut-NES2mut, $P < 0.0001$ and NES1mut-NES1/2mut, $P < 0.0001$). White boxes indicate regions shown as higher-magnification images. DNA was stained with Dapi. Scale bars, 10 µm. NES Nuclear Export Signal, AU arbitrary units, ns not significant.

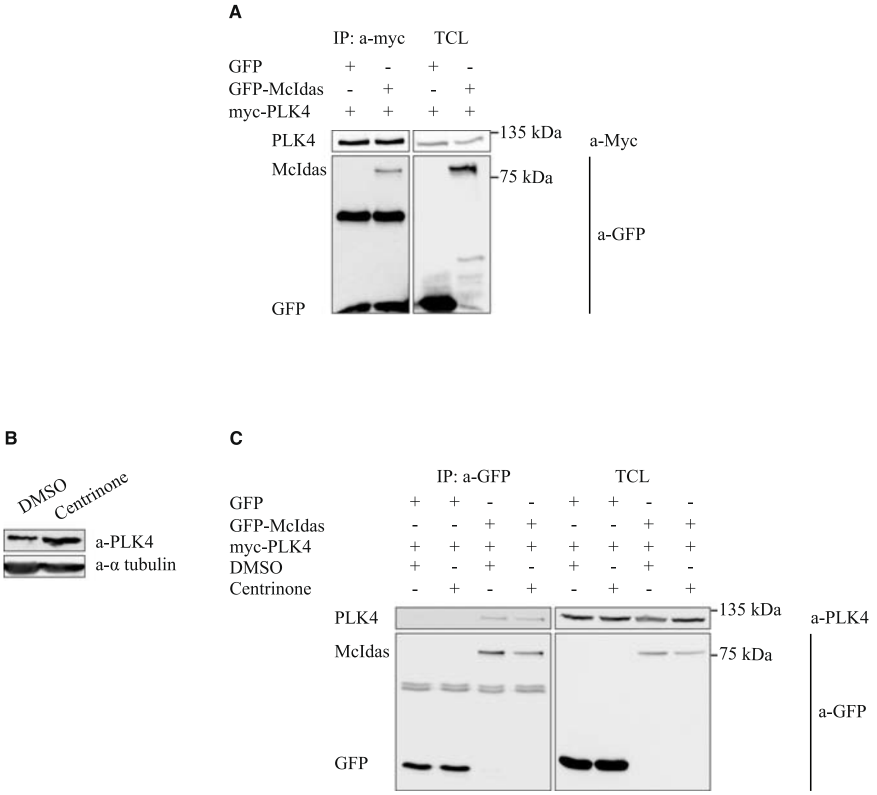

Figure EV5. McIdas interacts with PLK4 independently of PLK4 kinase activity.

(**A**) U2OS cells expressing myc-PLK4 under a tetracycline-dependent promoter were transfected with either GFP-tagged McIdas or GFP alone as a control. Immunoprecipitation was performed using an antibody against myc, and McIdas was detected in PLK4 immunoprecipitates. (**B**) U2OS cells expressing myc-PLK4 under a tetracycline-dependent promoter were cultured in the presence of 100 nM centrinone or DMSO as a control. Total cell lysates were analyzed for PLK4 expression. (**C**) U2OS cells expressing myc-PLK4 under a tetracycline-dependent promoter were transfected with either GFP-tagged McIdas or GFP alone as a control. After 48 h cells were cultured in the presence of 100 nM centrinone or DMSO as a control, and total protein lysates were collected. Immunoprecipitation was performed using an antibody against GFP. Centrinone-mediated inhibition of PLK4 kinase activity did not affect its interaction with McIdas.

 © The Author(s)

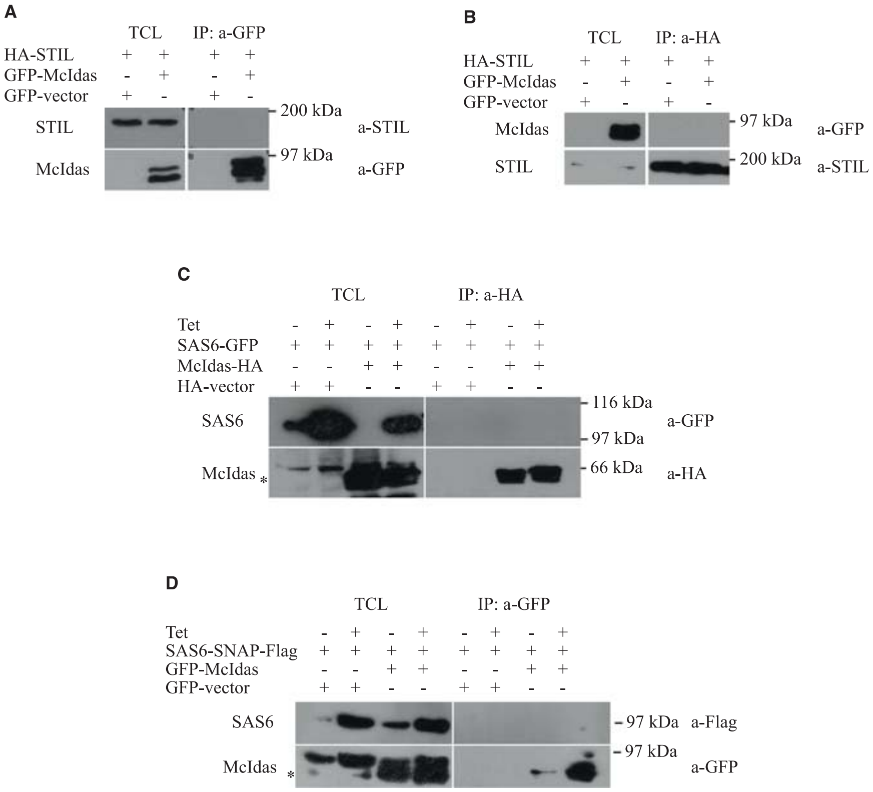

Figure EV6. McIdas is unable to interact with STIL and SAS6.

(**A**, **B**) HEK 293 T cells were co-transfected with vectors expressing HA-STIL and GFP-McIdas or GFP alone as a control. Cell extracts were collected and immunoprecipitation was performed using anti-GFP (**A**) or anti-HA (**B**) antibodies. STIL was not detected in McIdas immunoprecipitates and vice versa. (**C**) HEK 293 T cells were co-transfected with vectors expressing SAS6-GFP under a tetracycline-dependent promoter and either McIdas-HA or HA alone as a control. Cell extracts were collected and immunoprecipitation was performed using an anti-HA antibody. (**D**) HEK 293 T cells were co-transfected with vectors expressing SAS6-SNAP-Flag under a tetracycline-dependent promoter and either GFP-McIdas or GFP alone as a control. Cell extracts were collected and immunoprecipitation was performed using an antibody against GFP. No detectable interaction between McIdas and SAS6 was observed. Asterisks indicate the specific bands in each experiment. TCL total cell extract, IP immunoprecipitation.

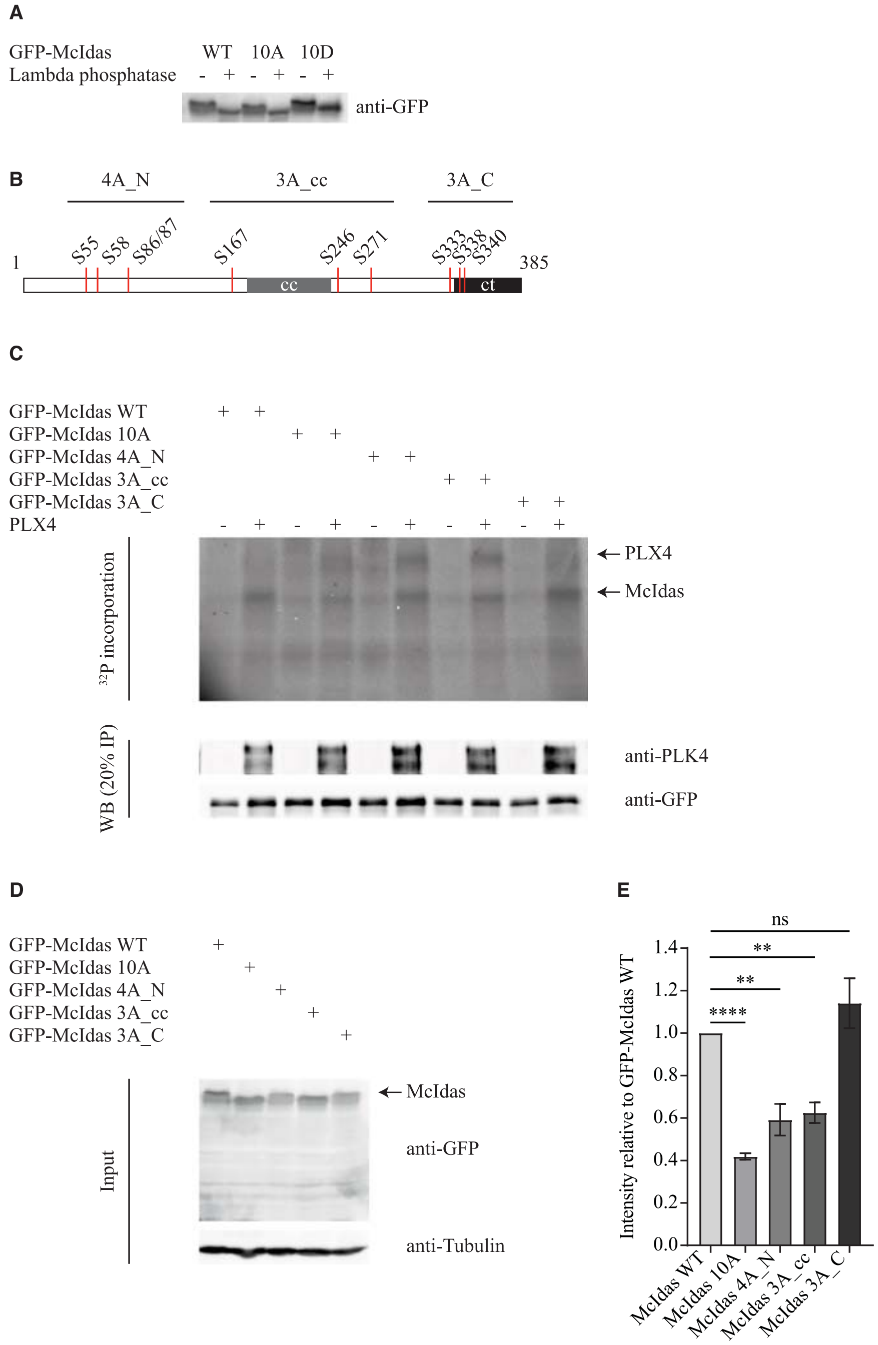

Figure EV7. PLK4-dependent phosphorylation of McIdas is enriched in its N-terminal and coiled coil-containing regions.

(**A**) HEK 293 T cells were transfected with vectors expressing GFP-McIdas WT or the mutants 10 A and 10D. Cell lysates were collected and incubated with lambda protein phosphatase. Western blot analysis was followed with an antibody against GFP. (**B**) Schematic representation of the PLK4-phosphorylated serine residues identified on the McIdas protein and the partial McIdas phospho-dead mutants generated. (**C**) In vitro kinase assays were performed using immunoprecipitated GFP-McIdas WT, GFP-McIdas 10 A or the indicated partial phospho-dead mutants. Each IP was incubated with recombinant Xenopus PLK4 (PLX4) and [γ-^{32}P] ATP. Samples were analyzed by autoradiography. Western blot analysis of 20% of each sample was performed using antibodies against PLK4 and GFP to verify protein loading. (**D**) Western blot analysis of total cell extracts used in (**C**) was performed with antibodies against GFP and α-tubulin. (**E**) Quantification of McIdas WT and mutant phosphorylation (from **C**) was performed using Image Lab software. Background signal in the control condition (no PLK4) was subtracted from the final intensity values. Values indicate phosphorylation signal intensity relative to McIdas WT (set as 1). Data are the mean values of three independent experiments and error bars indicate ± SEM. *P*-values were calculated using a two-tailed Student's t-test: **$P < 0.01$, ****$P < 0.0001$ and ns, not significant (*P* values: WT-10A, $P < 0.0001$; WT-4A_N, $P = 0.0054$; WT-3A_cc, $P = 0.0015$ and WT-3A_C: $P = 0.2988$).

 © The Author(s)

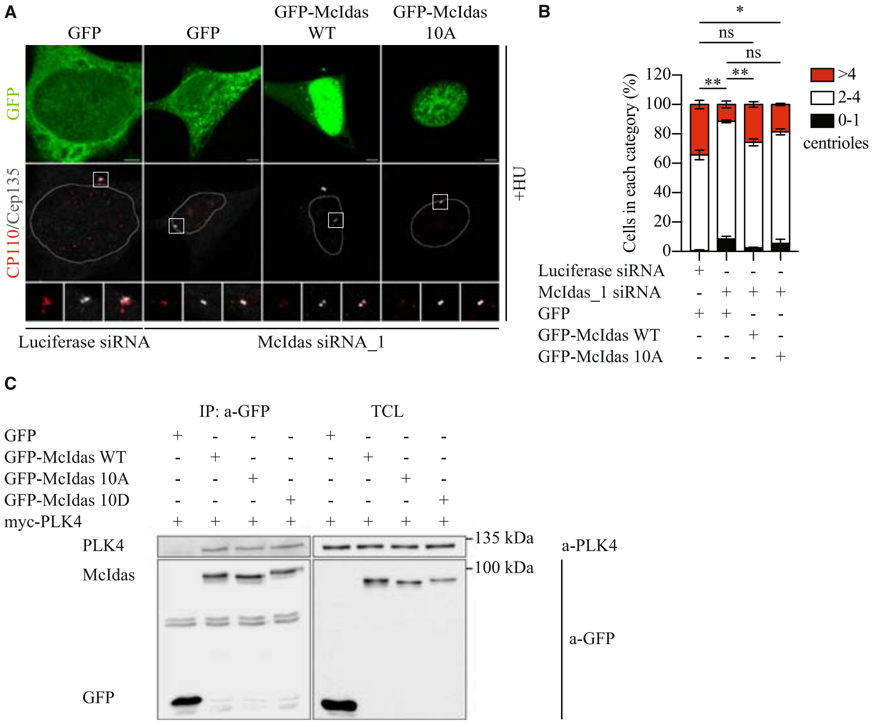

Figure EV8. PLK4-specific phosphorylation of McIdas is essential for daughter centriole biogenesis but is independent of their interaction.

(**A**) U2OS cells were transfected twice with McIdas siRNA_1 or control siRNA oligos. 24 h after the second siRNA transfection, cells were transfected with vectors expressing GFP-McIdas WT, GFP-McIdas 10A, or GFP alone as a control, followed by treatment with HU for an additional 48 h. Cells were fixed, and centriole numbers per cell were determined by staining with antibodies against Cep135 and CP110. (**B**) Quantification of centriole numbers per cell. The number of CP110 dots corresponds to the number of centrioles. Part of the immunofluorescence images in (**A**) and the corresponding quantification in (**B**) were also used in Fig. 2E and F, respectively, as part of the same experiment. Data are presented as the mean values of three independent experiments for GFP and GFP-McIdas WT or two for GFP-McIdas 10A and error bars indicate ± SEM. In each experiment at least 100 cells were counted per condition. Statistical values shown refer to the >4 centrioles category. *P*-values were calculated using two-tailed Student's t-test: **P* <0.1, ***P* <0.01 (*P* values: Luciferase siRNA/GFP-McIdas siRNA-GFP, *P* = 0.0035; McIdas siRNA/GFP-McIdas siRNA/McIdas WT, *P* = 0.0087; Luciferase siRNA/GFP-McIdas siRNA/McIdas WT, *P* = 0.0673, Luciferase siRNA/GFP-McIdas siRNA/McIdas 10 A, *P* = 0.0247 and McIdas siRNA/GFP-McIdas siRNA/McIdas 10 A, *P* = 0.0637. (**C**) GFP-tagged McIdas WT, GFP-McIdas 10 A, GFP-McIdas 10D, or GFP alone as a control were transfected into an inducible U2OS myc-PLK4 overexpressing cell line. Immunoprecipitation was performed using an antibody against GFP, and PLK4 was detected in immunoprecipitates of both McIdas WT and mutant proteins. Nuclear boundaries are outlined and white boxes indicate regions shown as higher-magnification images. Scale bars, 5 µm. HU hydroxyurea, ns not significant, TCL total cell extract, IP immunoprecipitation.

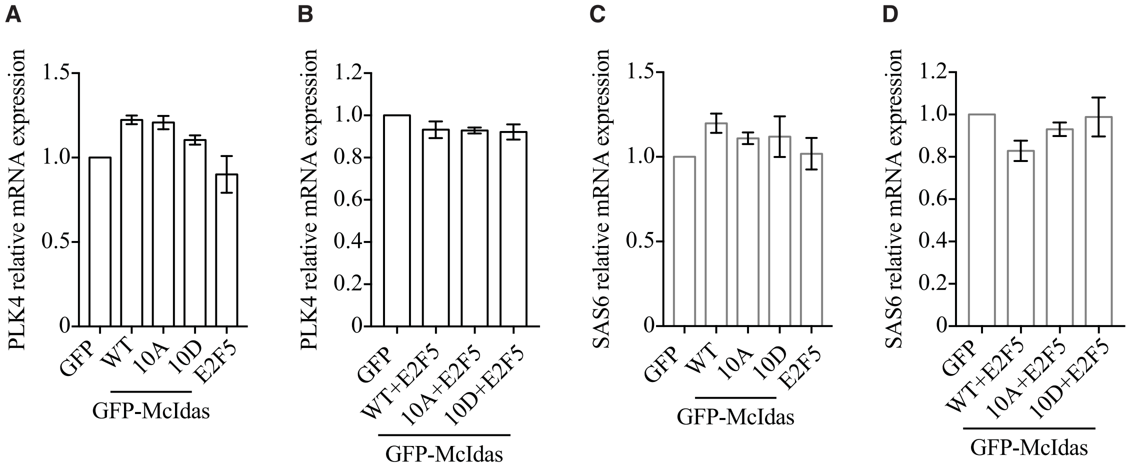

Figure EV9. PLK4 and SAS6 are not transcriptionally activated by McIdas.

(**A**, **B**) U2OS cells were transfected with vectors expressing the indicated genes and PLK4 mRNA expression was analyzed by quantitative real-time PCR (qPCR). (**C**, **D**) U2OS cells were transfected with the indicated genes and SAS6 mRNA expression was analyzed by quantitative real-time PCR (qPCR). Data are the mean values of at least three independent experiments and error bars indicate ± SEM.

 © The Author(s)
